# KTU-MEDAFE: A Newly Developed Multimodal Dataset for Emotion Recognition Using EEG–Speech Decision-Level Fusion

**DOI:** 10.3390/s26175608

**Published:** 2026-09-03

**Authors:** Bahar Hatipoglu Yilmaz, Betul Mumcu, Busra Ozkellekci

**Affiliations:** 1 Department of Computer Engineering, Engineering Faculty, Karadeniz Technical University, 61080 Trabzon, Türkiye; busraozkellekci@ktu.edu.tr; 2 Department of Software Engineering, Faculty of Technology, Karadeniz Technical University, 61080 Trabzon, Türkiye; betulmumcu@ktu.edu.tr

**Keywords:** affective computing, decision-level fusion, EEG-based emotion recognition, KTU-MEDAFE dataset, multimodal emotion recognition, speech emotion recognition

## Abstract

One of the central challenges in affective computing is achieving reliable emotion recognition for natural and effective human–computer interaction. In this study, we introduce KTU-MEDAFE (Karadeniz Technical University Multimodal Emotion Dataset using Audio, Facial Images, and EEG), a newly developed multimodal dataset containing synchronized EEG signals, speech recordings, and facial videos collected from 40 participants under controlled emotional elicitation conditions. The dataset includes Turkish emotional speech and two recording sessions conducted on separate days, providing a language-specific resource that supports both participant-dependent baseline evaluation and future session-separated analysis. Although KTU-MEDAFE comprises three modalities, the present study focuses on EEG and speech integration. EEG and speech recordings meeting signal quality criteria were transformed into image representations using the Angle–Amplitude Graph (AAG) method and classified using transfer learning with ResNet-50 and GoogLeNet architectures. To exploit complementary information across modalities, multiple decision-level fusion strategies were evaluated. Experimental findings show that multimodal fusion provides higher average classification performance than unimodal EEG and speech models across the evaluated binary emotion pairs, with performance varying according to subject, fusion strategy, and model architecture. Overall, the results support the potential benefit of combining EEG and speech for multimodal emotion recognition while highlighting substantial subject-dependent variability in classification performance.

## 1. Introduction

Emotions are a fundamental component of human communication, shaping social interaction, cognition, and decision-making. Automatic emotion recognition has therefore become a rapidly growing research area, particularly in the context of intelligent human computer interaction (HCI). Among different modalities such as facial expressions, gestures, and physiological signals, speech and visual cues stand out as the most widely utilized due to their naturalness and rich emotional content. As a result, multimodal emotion recognition (MER), which integrates multiple sources of information such as audio and video, has gained increasing attention in recent years because of its potential to improve robustness and capture complementary emotional information across modalities.

A review of the literature indicates that MER research has predominantly focused on feature extraction, classification, and fusion strategies. However, studies in which speech is treated as the primary modality, particularly when combined with physiological signals such as electroencephalography (EEG), remain limited. Furthermore, most existing approaches rely on benchmark datasets (e.g., eNTERFACE, IEMOCAP, RAVDESS, and RECOLA), which restrict generalizability due to language dependence, scripted scenarios, and culture-specific characteristics. In addition, subject-specific variability and limited linguistic diversity continue to pose challenges for robust MER. These limitations highlight the need for new datasets with broader linguistic and cultural coverage, as well as alternative fusion strategies to advance MER research.

In this study, we address several important limitations in MER by introducing the newly developed KTU-MEDAFE dataset, a multimodal emotion database specifically designed for controlled emotion analysis in Turkish. The dataset consists of recordings collected from 40 participants and includes three synchronized modalities recorded simultaneously under the same experimental conditions: EEG signals, speech recordings, and facial video recordings. An important characteristic of the proposed dataset is that the emotional speech recordings were collected in Turkish, thereby providing a language-specific multimodal resource that contributes to expanding linguistic diversity within MER research.

Although the complete KTU-MEDAFE dataset includes EEG, speech, and facial video modalities, the present study focuses on EEG–speech decision-level fusion (DLF) as an initial baseline evaluation. The facial video modality is not included in the current classification experiments because its effective use requires an additional facial preprocessing pipeline, including peak-frame or informative frame-sequence selection for each emotional trial. This modality will be incorporated in future work within a complete trimodal fusion framework.

To explicitly evaluate the contribution of multimodal integration, both unimodal and multimodal configurations were systematically examined. In the first stage, unimodal experiments were conducted independently for EEG and speech to establish baseline performance for each modality. For both modalities, the original signals were transformed into image-based representations prior to classification, allowing convolutional neural network (CNN) architectures to be applied within a unified processing framework. This unified representation also enables direct methodological comparison between modalities under identical classification conditions.

Subsequently, DLF strategies were applied to combine the outputs of the unimodal classifiers, enabling a direct and controlled analysis of performance gains achieved through multimodal integration. Within the scope of the proposed dataset and experimental framework, this study presents a systematic evaluation of EEG–speech MER using DLF. Since the proposed experimental framework involves multiple binary emotion pairs, several fusion strategies, five EEG channels, and two deep learning backbones, the overall computational burden becomes substantial when considering the full dataset. Therefore, experiments were conducted on recordings selected according to signal-quality and computational-feasibility criteria to ensure controlled evaluation while preserving subject diversity. This design enabled a controlled comparison between unimodal and multimodal approaches while maintaining stable model training and detailed subject-level evaluation. In addition, two distinct CNN architectures, ResNet-50 and GoogLeNet, were systematically evaluated under the same multimodal framework to investigate whether multimodal performance patterns remain consistent across different feature-extraction backbones.

Overall, the main contributions of this study can be summarized as follows: (i) introducing a newly developed Turkish multimodal emotion dataset containing synchronized EEG, speech, and facial recordings; (ii) constructing image-based representations for EEG and speech signals under a unified classification framework; (iii) systematically comparing unimodal and multimodal classification performances across multiple emotion pairs; (iv) examining conventional equal-weight, asymmetric weighted, and rank-based DLF formulations under subject-specific conditions; and (v) analyzing MER performance using two different deep learning architectures. In this way, the complementary roles of speech and EEG in MER are examined within a language-specific multimodal evaluation framework.

The remainder of this paper is organized as follows: Section 3 describes the proposed dataset in detail. Section 4 presents the experimental setup, including speech and EEG signal acquisition and preprocessing procedures. Section 5.1 and Section 5.2 describe the signal-to-image transformation and classification models, respectively. Section 5.3 explains the multimodal fusion strategies, Section 6 reports the experimental findings, and Section 7 concludes the paper.

## 2. Related Works

Early MER studies primarily focused on combining speech and facial information, as these modalities provide complementary affective cues and can be acquired non-invasively in human computer interaction (HCI) scenarios. Poria et al. [1] introduced a multimodal representation framework designed to extract and aggregate both semantic and emotional information from user-generated content in domains such as e-learning, e-health, and HCI. Tang et al. [2] evaluated MER using speech and video modalities on the eNTERFACE’05 and RAVDESS datasets through feature-level fusion (FLF). Nguyen et al. [3] proposed a framework combining Deep Belief Networks (DBNs) and 3D Convolutional Neural Networks (C3D), where DLF improved classification performance on the eNTERFACE dataset. Avots et al. [4] conducted a cross-corpus evaluation using Mel-frequency cepstral coefficients (MFCCs) as acoustic features and CNN-based visual features integrated at the decision level. Busso et al. [5] demonstrated that combining audio and facial modalities through both feature- and decision-level fusion significantly improves recognition accuracy. Garcia-Garcia et al. [6] introduced the Heterogeneous Emotional Results Aggregator (HERA), a technology-independent framework that integrates heterogeneous emotion recognition services into a unified architecture. Collectively, these studies show that audiovisual fusion has remained a dominant research direction because of its practical applicability and relatively mature benchmark resources.

Beyond audiovisual systems, studies have increasingly explored additional modalities and more diverse fusion strategies to improve robustness under different recording conditions and emotional contexts. Braunschweiler et al. [7] trained and analyzed deep learning models on multiple emotional speech corpora by incorporating speech, text, and their combinations. Using the IEMOCAP dataset, they proposed a deep learning architecture for MER that leverages both speech and text modalities. Yoon et al. [8] introduced a dual recurrent encoder model designed to jointly process speech and text for improved emotional dialogue understanding. Their framework encoded speech and text sequences using recurrent neural networks (RNNs) and fused them to predict emotion classes, with experiments conducted on the IEMOCAP dataset. Ortega et al. [9] proposed a fusion approach for continuous emotion recognition by integrating visual and auditory modalities in their respective representational spaces. Visual features were extracted using CNNs with transfer learning, and fusion was performed either at the feature level prior to training a single support vector regressor (SVR) or at the decision level after training separate SVRs for each modality. Experiments on the RECOLA dataset demonstrated that this approach benefited from the complementary information provided by visual and auditory modalities. Ringeval et al. [10] investigated machine learning methods for modeling contextual information to predict continuously annotated emotions, with experiments performed on the RECOLA dataset. Weber et al. [11] aimed to improve multimodal predictions by augmenting low-level features with higher-level geometry-based descriptors. Ding et al. [12] proposed a score-level fusion approach for emotion recognition based on facial expressions and speech. Facial features were extracted using a deep CNN pretrained on the FER2013 dataset, while speech emotion recognition was performed using long short-term memory recurrent neural networks (LSTM-RNNs). Yan et al. [13] presented a MER framework modeling three complementary cues, namely facial texture, facial landmarks, and speech signals, employing both feature-level and decision-level fusion. Their experiments were conducted on the AFEW and CHEAVD datasets. Tzirakis et al. [14] proposed a MER approach using auditory and visual modalities, where speech features were extracted using a pretrained CNN and visual features were derived from a deep residual network (ResNet). Their experiments were performed on the RECOLA dataset, which includes auditory, visual, and physiological recordings from 46 French-speaking participants. Sahoo and Routray [15] developed an audiovisual emotion recognition system that fused modalities at the decision level and evaluated performance on the Berlin EMODB, Assamese, and eNTERFACE’05 datasets. These contributions indicate that both feature-level and decision-level fusion remain actively used depending on modality characteristics and dataset structure.

Recent multimodal datasets and deep architectures have further expanded the field toward physiologically informed emotion analysis by incorporating physiological signals together with conventional audiovisual modalities. Wang et al. [16] introduced the Multimodal Emotion Database (MED4), which includes EEG, photoplethysmography (PPG), speech, and facial video recorded simultaneously from 32 participants under controlled emotional elicitation conditions. Lee et al. [17] proposed a multimodal emotion recognition framework integrating EEG with audio-visual features through contrastive learning and cross-modal attention, demonstrating the benefit of combining physiological EEG responses with audio-visual information for emotion recognition. More recently, Oh et al. [18] proposed an EEG–audio–video emotion recognition framework using modality-specific feature extraction, EEG-centered cross-attention, and contrastive learning, further demonstrating the complementary value of integrating neurophysiological and behavioral information. Dixit and Satapathy [19], Zhu et al. [20], and Li et al. [21] provide recent examples of deep multimodal architectures addressing real-time recognition, modality deficiency, and fine-grained feature integration in emotion recognition systems. Despite these advances, studies explicitly combining speech with EEG under subject-specific controlled conditions remain comparatively limited, particularly in language-specific multimodal datasets.

## 3. Dataset Description

To advance the analysis of human emotions in multimodal settings, we constructed an original dataset named the Karadeniz Technical University Multimodal Emotion Dataset using Audio, Facial Images, and EEG (KTU-MEDAFE). Unlike many existing datasets that focus solely on audio visual modalities, KTU-MEDAFE simultaneously incorporates speech, frontal facial video, and EEG signals, thereby providing a comprehensive foundation for MER research by enabling the joint evaluation of behavioral and physiological emotional responses under identical experimental conditions.

The dataset was recorded under controlled conditions at Karadeniz Technical University (KTU). A total of 40 participants, including undergraduate and graduate students, as well as faculty members, were recruited on a voluntary basis. All recordings were conducted in a controlled laboratory environment with minimized background noise following approval from the university’s ethics committee. To promote natural and consistent responses, participants were provided with detailed experimental instructions prior to data acquisition in order to minimize variability arising from recording conditions and task interpretation. Although KTU-MEDAFE was collected as a 40-participant multimodal dataset, the current classification experiments were conducted on a quality-controlled subset of 20 participants with complete, synchronized, and usable EEG–speech recordings. Participant S19 was excluded because the second recording session was not available, while participants S06, S11, S15 and S24 were excluded due to EEG signal quality problems in one or more sessions. In addition, after the recordings of the first 25 participants had been completed, a technical interruption in the EEG acquisition process temporarily affected the continuity of data collection. The remaining participant recordings were completed after the acquisition system became available again. However, the preprocessing, synchronization, and signal-to-image preparation of these later recordings could not be completed within the scope of the present manuscript. Therefore, the current experimental evaluation focuses on the initially processed and synchronized EEG–speech subset, while the remaining recordings will be incorporated in future extended studies using the complete dataset.

During the experimental sessions, participants were instructed to read text stimuli corresponding to four emotional categories: funny, sad, surprising, and neutral. When publicly available corpora containing suitable emotional texts were insufficient, additional textual materials were manually curated with the assistance of artificial intelligence applications. Specifically, funny texts consisted of jokes and humorous anecdotes, sad and surprising texts were derived from news reports and descriptions of social events and neutral texts were based on factual information devoid of emotional content. Because different text sets were used for the four emotion categories, the resulting speech recordings may also contain category-dependent lexical, phonetic, syntactic, and duration-related characteristics in addition to emotional prosody. These potential text-related effects should therefore be considered when using KTU-MEDAFE for speech-based emotion recognition. All stimuli and presentation procedures were standardized across participants to ensure consistent experimental conditions. Table 1 presents an overview of the dataset characteristics, whereas Table 2 details the structure of the experimental text stimuli.

**Figure 1:**
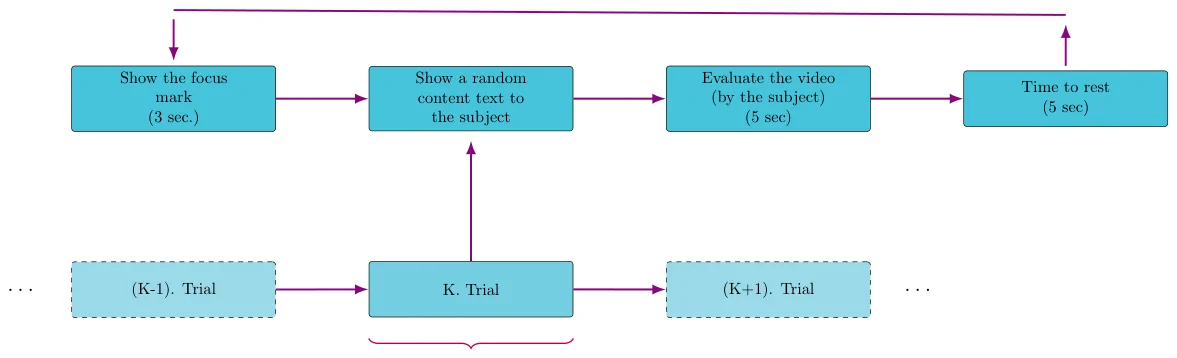
Flow chart of the experimental procedure.

Each trial followed a fixed experimental paradigm. As illustrated in Figure 1, every recording began with a brief resting phase. At t=0 s, a visual focus marker accompanied by an audible alert was presented, followed by a three-second countdown to enhance participant concentration. Subsequently, a randomly selected text stimulus was displayed on the screen, which participants read aloud while multimodal data—including speech, facial video, and EEG—were recorded simultaneously. At the conclusion of each trial, an audible alert signaled the end of the recording, after which the screen turned black and participants were prompted to rate their perceived emotional state on a scale ranging from 1 to 9.

To examine the validity of the intended emotional labels, we analyzed the self-assessment scores collected from the participants in both paradigms. The original stimulus labels were coded as neutral = 1, funny = 2, surprising = 3, and sad = 4. Consistent with the participant set used in the current experimental evaluation, S06, S11, S15, S19 and S24 were excluded from this analysis. Therefore, the analysis was conducted using 20 participants. The analysis was performed separately for the first and second paradigms, and the results are presented in Table 3 and Table 4, respectively. Each paradigm included 172 trials, with 43 trials for each emotional category. Thus, 860 participant-level ratings were analyzed for each emotional class and 3440 ratings were analyzed in total for each paradigm.

As shown in Table 3 and Table 4, the overall mean self-assessment scores were 6.57 ± 2.20 for the first paradigm and 6.53 ± 2.18 for the second paradigm on a 1–9 scale. In both paradigms, the neutral category obtained the highest mean ratings, with 88.15% and 90.59% of the ratings being equal to or higher than 6 in the first and second paradigms, respectively. The sad and surprising categories also showed relatively strong agreement with the intended labels in both paradigms, with mean scores above 6.60. In contrast, the funny category received lower mean scores than the other categories, with 5.29 ± 2.42 in the first paradigm and 4.96 ± 2.29 in the second paradigm.

Each participant completed two recording sessions conducted on separate days to enable future session-separated evaluation and the investigation of session-related variability. In each session, 172 recordings per participant were collected across the four emotion categories, resulting in a total of 344 speech signals, 344 facial videos, and 344 EEG recordings per participant. Although the present study uses a participant-dependent session-mixed protocol as an initial baseline, the two-session structure of KTU-MEDAFE enables strict session-to-session training and testing in future studies. Such an evaluation provides a more challenging setting for assessing cross-session robustness and reducing performance overestimation associated with session-mixed evaluation.

For EEG acquisition, the Emotiv Insight 2 Brainwear headset was employed because of its ease of setup and portability. All modalities were acquired using standardized recording settings and stored in high-quality formats to support reproducibility and subsequent analysis.

Among currently available multimodal emotion datasets, KTU-MEDAFE provides a rare combination of synchronized speech, facial video, and EEG recordings collected under controlled text-reading conditions in Turkish. This structure enables direct comparison of physiological and behavioral emotional responses obtained from the same elicitation process under identical temporal conditions. The combination of synchronized multimodal acquisition, carefully designed stimuli, and systematic labeling makes KTU-MEDAFE a valuable resource for MER research, particularly by addressing linguistic diversity and supporting future controlled session-based and subject-dependent multimodal analyses. Detailed statistics of the dataset are provided in Table 1, and the experimental flow paradigm is illustrated in Figure 1.

The relatively lower and more variable self-assessment scores observed for the funny category indicate that humorous stimuli did not elicit the intended emotional response equally across all participants. This finding is consistent with the highly subjective nature of humor perception and should be considered when using the funny category in subsequent analyses. Accordingly, the participant-level self-assessment scores are provided to enable researchers to apply trial-selection criteria according to the objectives of their studies.

## 4. Experimental Setup

### 4.1. Speech Acquisition and Processing

Prior to constructing the proposed dataset, speech acquisition and preprocessing procedures reported in related studies were examined. In [1], speech was recorded as part of a multimodal framework combining text, audio, and video, with one of the employed corpora being the eNTERFACE dataset, sampled at 48 kHz with a bitrate of 1536 kbps. Grimm et al. [22] downsampled audio recordings to 16 kHz with a 16-bit resolution. Engberg et al. [23] employed a high-quality microphone, applied a 5 Hz high-pass filter to suppress low-frequency noise, and recorded speech signals at a sampling rate of 48 kHz. Busso et al. [24] used two high-quality shotgun microphones directed at each participant to synchronously capture speech at 48 kHz. McKeown et al. [25] combined wearable microphones with room microphones, using the former as the primary audio source and the latter for background noise modeling. Their recordings were acquired at 48 kHz with a 24-bit resolution.

In the present study, speech was recorded in a controlled laboratory environment using a pre-polarized condenser microphone with a cardioid polar pattern. The microphone provides a frequency response ranging from 20 Hz to 20 kHz and supports a 24-bit analog-to-digital (A/D) resolution at a sampling rate of 48 kHz, enabling the high-fidelity capture of acoustic signals. The microphone also supports a maximum sound pressure level (SPL) of 125 dB at maximum gain and 85 dB at minimum gain, allowing accurate recording across a wide dynamic range. All recordings were performed via a Universal Serial Bus Type-C (USB-C) connection, with the microphone powered directly through the USB bus. Audio data acquisition was carried out using Audacity, which enabled the reliable recording and storage of the raw signals. Following acquisition, the recorded speech signals were transferred to a dedicated workstation for subsequent processing and analysis.

To maintain consistency across subjects, all recordings were stored using identical acquisition settings without altering the original sampling frequency or bit resolution during the initial recording stage. During preprocessing, each speech sample was segmented according to the trial boundaries defined by the experimental protocol, ensuring temporal correspondence with the EEG and facial recordings. This alignment was particularly important for the subsequent multimodal fusion experiments, as all modalities had to correspond to the same emotional trial under synchronized acquisition conditions. No additional filtering or feature-specific preprocessing was applied at this stage, thereby preserving the original acoustic structure prior to signal-to-image transformation.

To further characterize the potential influence of text-dependent properties on the speech recordings, utterance duration and local-maxima counts were analyzed separately for the two paradigms using all speech recordings from the 20 participants included in the present experiments. Each emotion category comprised 860 utterances per paradigm (20 participants × 43 utterances). Utterance duration was calculated directly as the number of audio samples divided by the corresponding sampling frequency.

As summarized in Table 5, the mean utterance durations were relatively close across categories. In the first paradigm, they ranged from 10.08 ± 1.51 s to 10.81 ± 1.61 s, while in the second paradigm they ranged from 10.30 ± 1.59 s to 11.34 ± 1.90 s. The funny category showed a somewhat longer mean duration in the second paradigm; nevertheless, the category distributions exhibited substantial within-category variability and overlap. A similar descriptive pattern was observed for the local-maxima counts, with no pronounced category-wise separation at the descriptive level.

Importantly, the signal-to-image transformation does not use utterance duration or the absolute number of local maxima as explicit classification features. Instead, each local maximum is characterized according to its local geometric relationship with neighboring minima, and the resulting distance-based descriptors are mapped onto a fixed two-dimensional image plane. Thus, the relationship between signal duration, local-maxima count, and the final image representation is indirect rather than one-to-one; local maxima exhibiting similar geometric relationships may be represented in the same or neighboring regions of the image domain. Nevertheless, because category-specific texts were used, lexical, phonetic, syntactic, and duration-related effects cannot be completely excluded and should be considered when interpreting the speech-based results.

### 4.2. EEG Acquisition and Processing

EEG recordings were acquired using the Emotiv Insight 2 headset, which provides five active channels positioned at AF3, AF4, T7, T8, and Pz according to the international 10–20 system. The EEG signals acquired from these electrodes were used throughout the study and were denoted as channels Ch1–Ch5, respectively. Common mode sense/driven right leg (CMS/DRL) reference electrodes were placed on the left mastoid. The device records EEG signals at a sampling rate of 128 Hz, with an internal sampling rate of 2048 Hz, and provides a resolution of 14 bits. The system operates within a bandwidth of 0.5–43 Hz and incorporates digital notch filters at 50 Hz and 60 Hz, along with a built-in fifth-order Sinc filter as part of the device-side signal-conditioning pipeline. These specifications were considered sufficient for capturing emotion-related EEG activity, particularly within frontal and temporal regions commonly associated with affective processing. The device was selected not only because of its portability and ease of application, but also because its five-channel configuration allows stable data acquisition under controlled multimodal recording conditions. Data transmission is achieved via a proprietary 2.4 GHz wireless connection, and the device is powered by a lithium-polymer (LiPo) battery with a typical operating time of up to 8 h. To monitor signal quality, the headset supports real-time impedance assessment through a patented contact quality system. The electrodes are composed of semi-dry polymer material, supporting stable recordings over extended sessions. All EEG data were acquired using EmotivPRO software, which enabled real-time monitoring, acquisition, and storage of the signals. Following acquisition, the recorded data were transferred to a dedicated workstation for further preprocessing and analysis.

Prior to finalizing the EEG acquisition protocol, a comprehensive review of existing MER datasets was conducted to inform the selection of sampling rates, electrode locations, and filtering strategies. Zheng and Lu [26], for example, recorded EEG data from 15 participants at 200 Hz and applied a 0.3–50 Hz band-pass filter to reduce noise and artifacts. Similarly, Chao et al. [27] utilized the DEAP dataset, originally sampled at 512 Hz, but downsampled the signals to 128 Hz for subsequent analysis. Wichakam and Vateekul [28] also employed the DEAP dataset but selected only ten channels (F3, F4, Fp1, Fp2, P3, P4, T7, T8, O1, and O2) to focus on emotion-relevant regions. Taran and Bajaj [29] introduced a custom dataset collected from 20 subjects using 24 EEG channels sampled at 256 Hz, emphasizing the role of frontal and temporal electrodes in emotional processing. Nakisa et al. [30] reported the MAHNOB-HCI dataset, which provides 32-channel EEG recordings at 256 Hz. Additional studies have employed the 14-channel Emotiv EPOC device at 128 Hz [31] and 24-channel systems sampled at 256 Hz [32], consistently indicating that the most informative EEG features for emotion recognition are predominantly concentrated below 40–45 Hz. Accordingly, the selected acquisition and preprocessing parameters were chosen to preserve emotion-relevant EEG frequency components while maintaining compatibility with mobile EEG-based experimental settings.

Based on these considerations, a band-pass filter between 4 and 45 Hz was applied to the recorded EEG signals to isolate frequency components relevant to emotional states while suppressing low-frequency drifts and high-frequency noise. All preprocessing steps were performed using the EEGLAB toolbox within MATLAB R2025b. Raw EEG data were exported from EmotivPRO in comma-separated values (CSV) format, imported into MATLAB, and subsequently converted into EEGLAB’s .set format for further analysis. The same preprocessing pipeline was applied consistently across all subjects, channels, and emotional categories to ensure comparability of the generated EEG signal-image representations. The selected filtering range was chosen in accordance with established practices in emotion recognition research.

Although the selected acquisition and filtering settings are consistent with commonly employed EEG-based emotion-recognition preprocessing strategies, band-pass and device-side filtering alone cannot guarantee the complete removal of speech-related myogenic and ocular activity. Since EEG was recorded while participants read the stimuli aloud, residual activity associated with facial and temporal muscles, jaw movement, and eye movement may remain in the EEG signals. Artifact rejection based on independent component analysis (ICA) was not applied in the present study. Although ICA can technically be used with five-channel EEG recordings, the low channel density and spatial distribution of the available electrodes limit the number and interpretability of independent components, making reliable retrospective separation of neural, ocular, and muscular sources challenging. Therefore, the EEG results reported in this study should be interpreted as participant-dependent baseline measurements acquired during overt speech rather than as representations containing exclusively neural activity.

## 5. Methods

### 5.1. Signal-to-Image Transformation Using Local Maxima

The signal-to-image transformation used in this study is derived from the Angle–Amplitude Graph (AAG) representation introduced in our previous work [33]. In the original formulation, the local maxima and minima of a one-dimensional signal were changing points, and the representation was constructed from two geometric quantities: the left and right Euclidean distances between neighboring changing points and the angles formed by these distances. The resulting angle and normalized amplitude values were subsequently mapped onto a two-dimensional image plane. Importantly, the original method was formulated at the level of the time-domain waveform and was introduced as a signal-to-image transformation applicable to arbitrary time-domain signals. In the present study, this formulation is adapted to provide a more compact representation by retaining only the distance-based information associated with local maximum points. This modified formulation is applied to both EEG and speech signals, thereby providing a common signal-to-image representation for the two modalities.

Let a discrete time-series signal be represented as $S\;=\;{\left\{\left(x_{n},y_{n}\right)\right\}}_{n=1}^{N}$, where $x_{n}$ denotes the temporal coordinate and $y_{n}$ denotes the amplitude value of the signal. A point is considered a local maximum if it represents a locally dominant amplitude within its neighborhood, without necessarily being the global maximum of the signal. After detecting all local maxima and minima, each local maximum point ${max}_{i}\left(x_{i},y_{i}\right)$ is associated with its neighboring local minimum points on the left and right, denoted as ${min}_{k}\left(x_{k},y_{k}\right),\min_{k+1}\left(x_{k+1},y_{k+1}\right)$.

For each local maximum region, the Euclidean distances between the maximum point and its neighboring minimum points are computed. The left-side distance for the *i*-th maximum point is defined in Equation (1), where $X_{i,k}\;=\;x_{i}-x_{k},Y_{i,k}\;=\;y_{i}-y_{k}$:

(1) $$a_{i}\left(t_{i}\right)\;=\;\sqrt{{\left(X_{i,k}\right)}^{2}+{\left(Y_{i,k}\right)}^{2}}$$

Similarly, the right-side distance for the same maximum point is calculated as defined in Equation (2), where $X_{i,k+1}\;=\;x_{i}-x_{k+1},Y_{i,k+1}\;=\;y_{i}-y_{k+1}$:

(2) $$b_{i}\left(t_{i}\right)\;=\;\sqrt{{\left(X_{i,k+1}\right)}^{2}+{\left(Y_{i,k+1}\right)}^{2}}$$

These distances characterize the geometric configuration of the signal around each local maximum and constitute the primary descriptors used in the transformation process. To obtain a normalized structural measure, the relative magnitude between the left and right distances is evaluated. The normalized amplitude descriptor corresponding to each maximum point is computed as shown in Equation (3):

(3) $$R_{i}\left(t_{i}\right)=\left\{\begin{array}{cc} \frac{b_{i}\left(t_{i}\right)}{a_{i}\left(t_{i}\right)}, & b_{i}\left(t_{i}\right)<a_{i}\left(t_{i}\right)\\ -\frac{a_{i}\left(t_{i}\right)}{b_{i}\left(t_{i}\right)}, & \mathrm{otherwise} \end{array}\right.$$

This normalization ensures scale consistency while preserving the directional relationship between neighboring structures around each maximum point. Each detected maximum point contributes to a two-dimensional representation constructed from these distance-based descriptors. The transformed image representation of a signal is obtained by placing all computed values into a structured image plane as $\mathrm{Img}\left(u,v\right)\;=\;\sum_{i=1}^{n}{\mathrm{Img}}_{i}\left(R_{i}\left(t_{i}\right)\right)$, where *n* denotes the number of detected local maximum points and ${\mathrm{Img}}_{i}$ represents the mapping of the descriptor associated with the *i*-th local maximum into the image domain.

By constructing the representation using only local maximum-based distance measures, the proposed formulation reduces representational redundancy while preserving dominant morphological characteristics of the signal.

**Figure 2:**
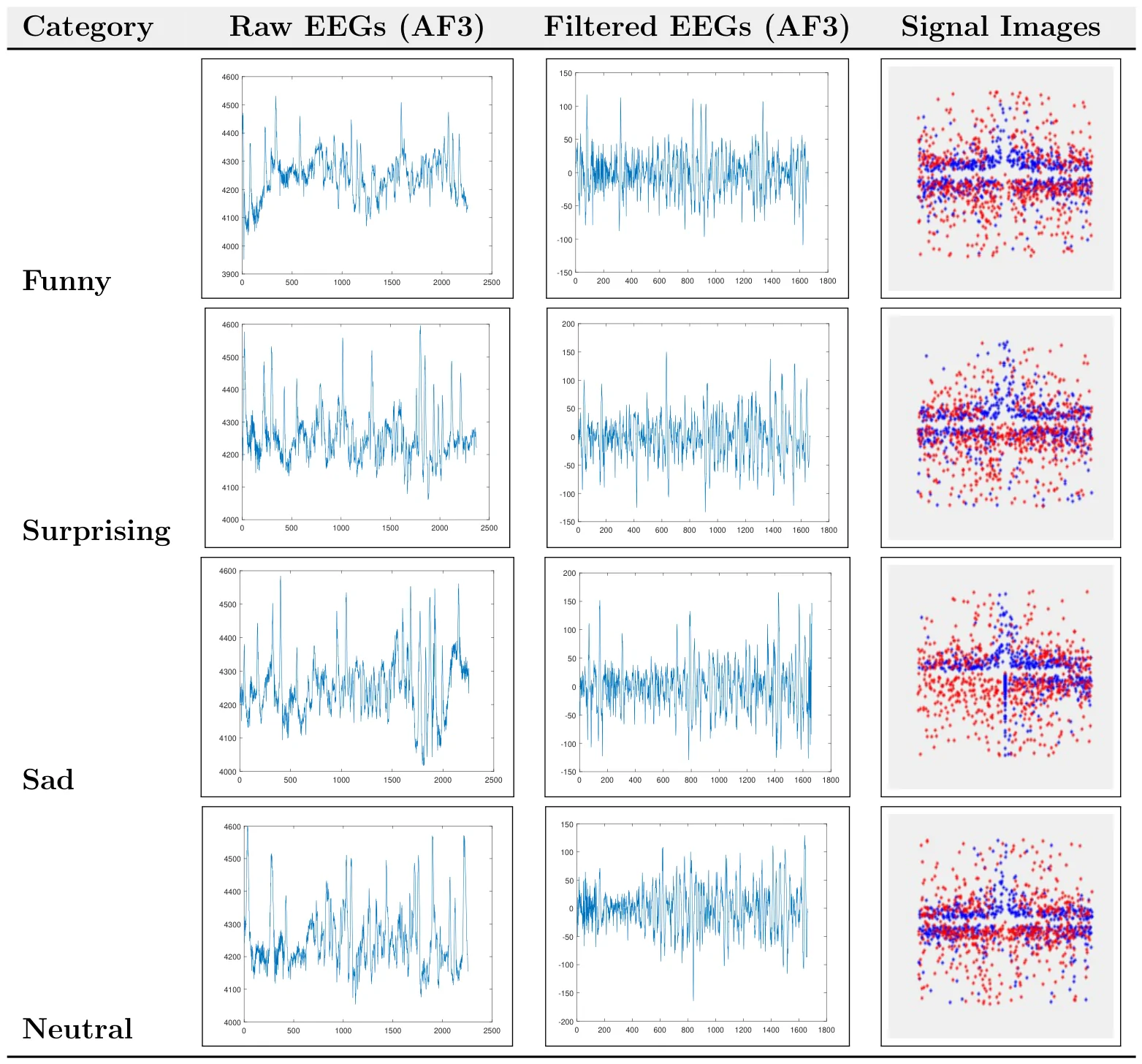
Raw EEG signals recorded from a subject while reading text stimuli from four emotional categories (**left**), their filtered versions after preprocessing (**center**), and the corresponding image representations generated by the proposed signal-to-image transformation (**right**). In the image representations, blue markers indicate the angle–amplitude points obtained from the lines connecting each maximum to the first minimum, whereas red markers represent the second-distance points.

**Figure 3:**
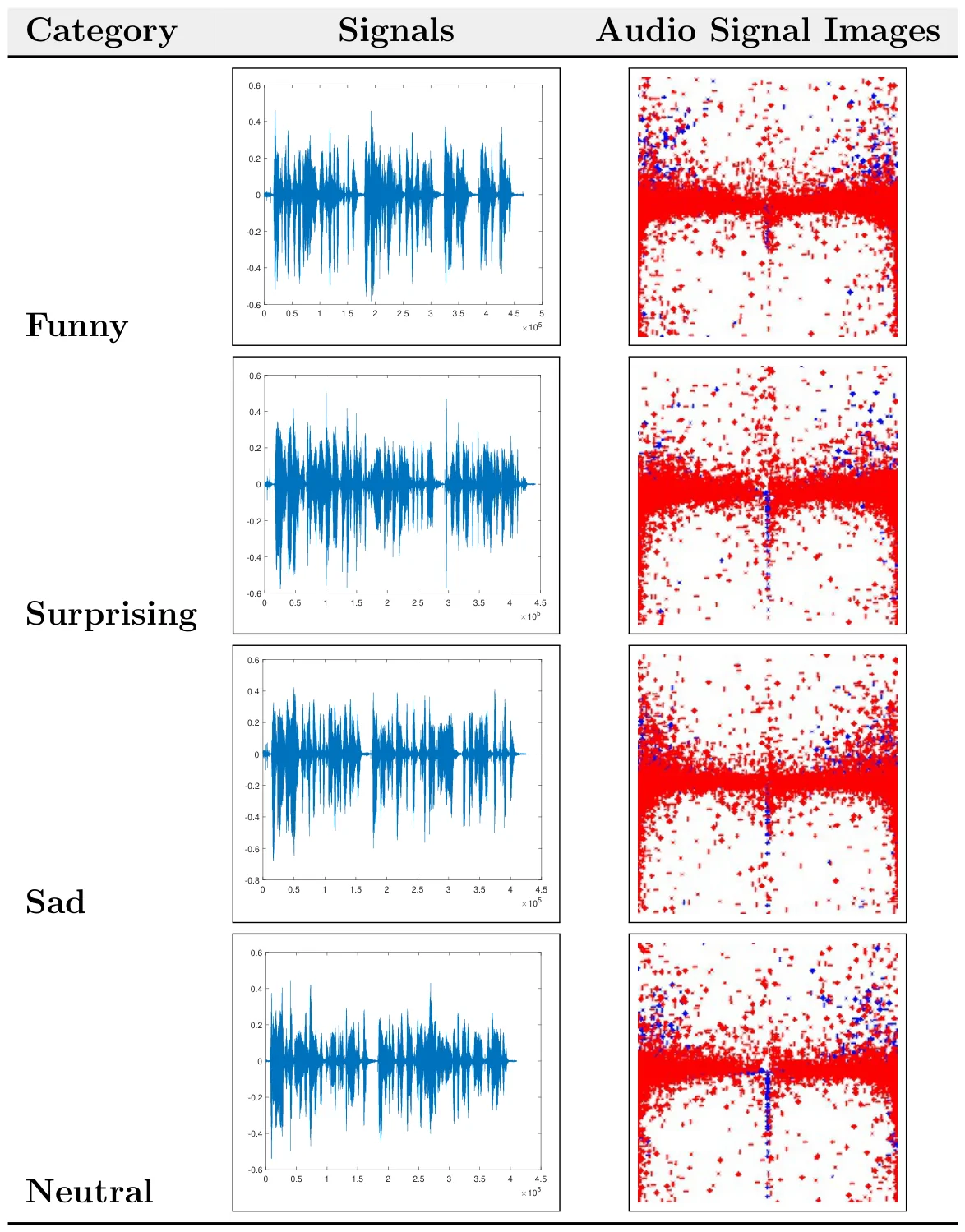
Audio signals recorded from a subject while reading text stimuli from four emotional categories (**left**) and the corresponding image representations generated by the proposed signal-to-image transformation (**right**).

The proposed distance-based transformation was applied to both EEG signals (Figure 2) and speech signals (Figure 3) contained in the dataset. By converting these one-dimensional signals into a unified image-based representation using the same local-maximum distance formulation, structurally comparable visual features were obtained across modalities. This common representation space enabled the integration of the generated images within a multimodal framework, allowing the effective multimodal fusion of EEG- and speech-derived signal images. Consequently, complementary information from both physiological and acoustic modalities was jointly exploited during the learning process, improving the overall discriminative capability of the proposed system.

From the EEG perspective, the use of this representation is supported by our previous study [33], in which the original AAG transformation was experimentally evaluated using both EEG and magnetoencephalography (MEG) signals and demonstrated effective classification performance. These findings established the feasibility of representing neurophysiological time series signals through the geometric relationships between successive changing points. In the present study, the same underlying principle is retained for the EEG branch of KTU-MEDAFE, while the reduced distance-based formulation provides a more compact representation of local EEG waveform variations.

From the speech perspective, speech recordings are likewise one-dimensional, time-varying signals containing successive local maxima and minima. In a speech waveform, the geometric relationships between neighboring extrema reflect local variations in waveform amplitude together with their temporal spacing. Therefore, the same signal-level geometric principle can be applied to speech without requiring EEG-specific signal characteristics. Although EEG and speech originate from fundamentally different physiological processes, the transformation is applied here as a unified computational representation of local waveform geometry rather than as an indication that the two modalities have identical physical interpretations. This common representation enables EEG- and speech-derived images to be processed using the same ResNet-50 and GoogLeNet classification framework and subsequently integrated at the decision level.

### 5.2. Classification

In this study, a subject-dependent classification framework was adopted for 20 participants with complete and synchronized EEG–speech recordings. For each participant, EEG and speech samples were paired according to their corresponding recording identifiers. Samples obtained from both recording sessions were then merged and divided into training (70%), validation (10%), and test (20%) sets using stratified random sampling to preserve the class distribution. The same split indices were applied to the paired EEG and speech samples to maintain sample-level correspondence for multimodal fusion. A fixed random seed was set before data partitioning to ensure reproducibility and to maintain identical data partitions across the corresponding EEG and speech experiments.

Accordingly, the experiments followed a participant-dependent, session-mixed 70/10/20 evaluation protocol rather than a strict session-based or subject-independent evaluation. This design was selected because the amount of data available for each participant was limited, and a strict session-based split would have substantially reduced the training data available for transfer learning. Therefore, the reported results should be interpreted as participant-dependent baseline performance rather than as evidence of session-independent or subject-independent generalization. Pairwise classification was performed for the six emotion combinations: 1 vs. 2, 1 vs. 3, 1 vs. 4, 2 vs. 3, 2 vs. 4, and 3 vs. 4.

The AAG representations derived from EEG and speech signals were used to train separate ResNet-50 and GoogLeNet models initialized with ImageNet weights. For each modality, the original classification head of the pre-trained network was replaced with a task-specific fully connected layer, followed by softmax and classification layers. The networks were fine-tuned end-to-end using the EEG- and speech-derived AAG images. To facilitate adaptation of the newly added classification head, the weight and bias learning-rate factors of the new fully connected layer were set to 10.

Although AAG representations differ substantially from the natural images used for ImageNet pre-training, the use of pre-trained networks in this study does not assume semantic similarity between the two image domains. Instead, ImageNet-pretrained networks were used as initialization for transfer learning under the limited-sample, participant-dependent setting. ImageNet-pretrained CNNs learn low- and intermediate-level visual patterns, such as edges, local contrast variations, textures, and spatial structures, which may also provide transferable information for structured non-natural image representations. Previous studies have similarly demonstrated the effectiveness of ImageNet-pretrained CNNs for non-natural two-dimensional inputs such as audio spectrograms [34]. Therefore, ImageNet pre-training was used primarily as a data-efficient initialization strategy that may reduce the risk of overfitting compared with training the networks entirely from random initialization, rather than as a means of transferring natural-image object semantics to the AAG domain.

Data augmentation was applied only to the training images to improve model robustness. The augmentation procedure included random horizontal reflection and random rotations between −10° and +10°. The same augmentation strategy was applied to both EEG- and speech-derived AAG images. No augmentation was applied to the validation or test sets. These images were only resized to the input dimensions required by the corresponding backbone network.

The models were optimized using the Adam optimizer. EEG and speech networks were trained for a maximum of 50 and 70 epochs, respectively, with the training data shuffled at every epoch. A grid search was conducted over mini-batch sizes of 8, 16, and 32; EEG learning rates of 5 × 10^−4^ and 1 × 10^−4^; and speech learning rates of 3 × 10^−4^ and 1 × 10^−4^.

For each candidate configuration, posterior class probabilities were obtained from the corresponding EEG and speech models on the validation set and combined using the sum, product, max, weighted-sum, and Borda-count fusion rules. For weighted-sum fusion, α values of 0.3, 0.5, and 0.7 were evaluated, where α represents the contribution of the EEG modality and 1−α represents the contribution of the speech modality. For each subject and emotion pair, the training hyperparameters, EEG channel, fusion rule, and weighting coefficient, when applicable, were selected exclusively according to validation fusion accuracy. The held-out test set was not used for hyperparameter, model, or fusion-configuration selection and was accessed only after completion of the validation-based selection procedure.

The configurations selected on the validation set were subsequently evaluated on the held-out test set, and the corresponding unimodal EEG, unimodal speech, and multimodal fusion performances were reported as the final test results.

#### 5.2.1. ResNet-50

**Figure 4:**
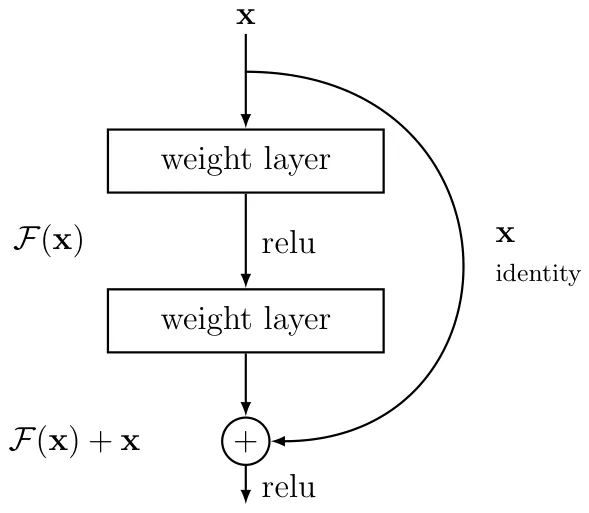
Residual learning block illustrating shortcut connections in ResNet.

ResNet, proposed by He et al. [35], is a CNN architecture designed to address the degradation problem that arises as network depth increases, making the optimization of deep neural networks more difficult. In the ResNet architecture, instead of directly learning the desired mapping, layers are designed to learn residual functions. This approach is known as residual learning. As shown in Figure 4, this is accomplished through shortcut connections that add the input directly to the output, bypassing one or more layers. Identity shortcut connections facilitate gradient propagation across the network without introducing additional parameters.

The ResNet-50 architecture used in this study is based on a bottleneck block design that allows network depth to be increased while keeping computational cost relatively low. A bottleneck block consists of a sequence of 1 × 1 convolutions that reduce the channel size, a 3 × 3 convolution that performs feature extraction, and a final 1 × 1 convolution that recovers the channel size. In this design, the 3 × 3 convolution operates in a lower-dimensional feature space, increasing parameter and computational efficiency. Projection shortcuts based on 1 × 1 convolutions are used when the spatial or channel dimensions of the feature maps change, while parameterless identity connections are preferred when dimensions are preserved.

**Figure 5:**
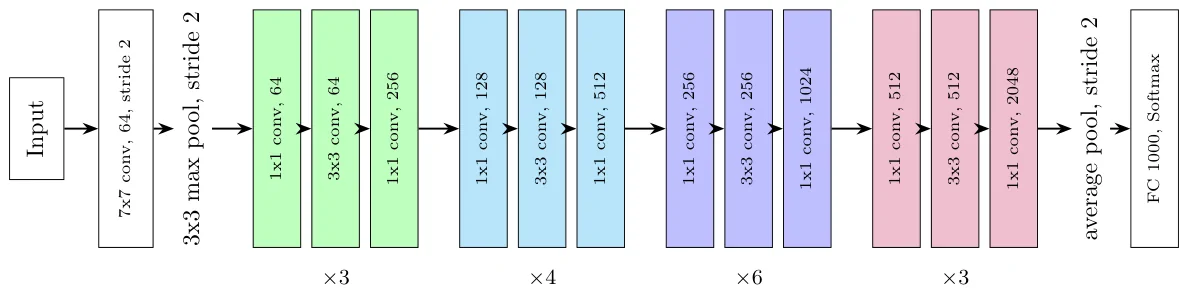
Overall architecture of the ResNet-50 model used in the classification experiments.

The overall structure of ResNet-50 comprises an initial 7 × 7 convolutional layer with a stride of 2, followed by a total of 16 bottleneck blocks grouped into four main stages, and finally a global average pooling (GAP) layer and a fully connected layer for classification. The ResNet-50 model, whose architecture is depicted in Figure 5, contains a total of 50 weight layers and has an approximate computational complexity of 3.8 billion FLOPs. Compared with relatively shallower architectures such as VGG-19, ResNet-50 achieves a lower computational cost despite being a deeper network. Furthermore, in accordance with the original design of the architecture, batch normalization (BN) is applied after each convolutional layer and before the ReLU activation function, facilitating the optimization of the deep network.

#### 5.2.2. GoogLeNet

**Figure 6:**
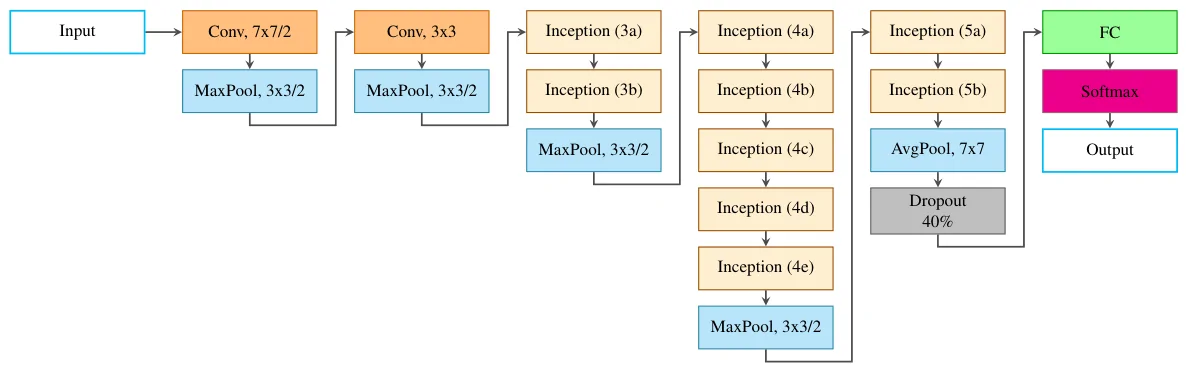
Overall architecture of the GoogLeNet model used in the classification experiments.

GoogLeNet is a deep CNN architecture that achieved state-of-the-art performance in the image classification and object detection tasks of the ImageNet Large Scale Visual Recognition Challenge (ILSVRC) 2014. The overall layer-wise structure of the GoogLeNet architecture is presented in Figure 6.

**Figure 7:**
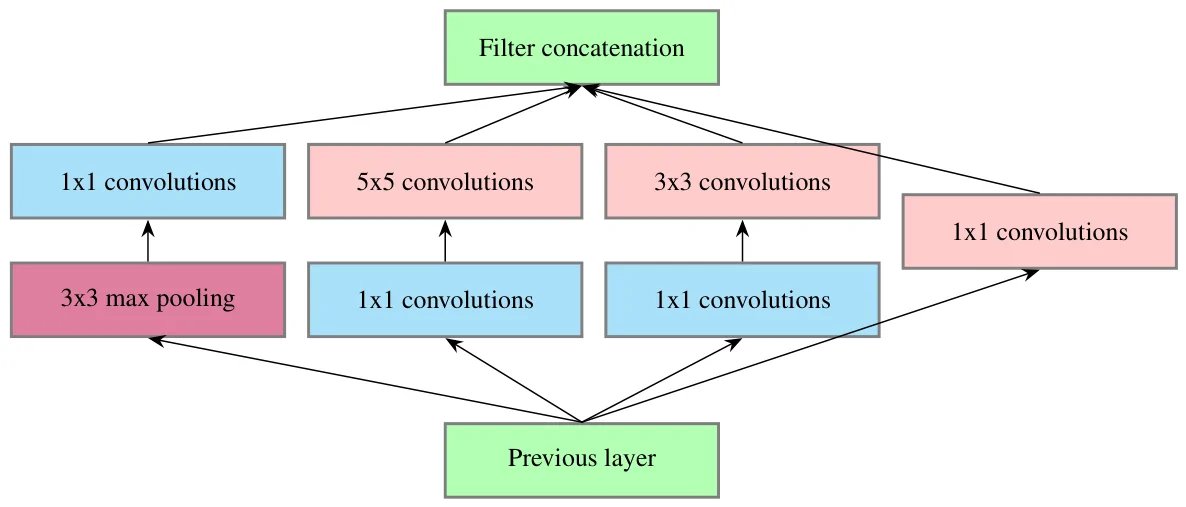
Parallel multi-scale structure of the Inception module.

This model was introduced by Szegedy et al. [36] as a 22-layer implementation of the Inception approach, designed to improve computational efficiency while maintaining strong representational capacity in deep CNNs. The fundamental building block of this architecture is the Inception module. The parallel branching structure and multi-scale filter organization of the Inception module are illustrated in Figure 7. This module adopts a multi-scale processing strategy to capture visual features at different spatial scales. Unlike conventional sequential layer structures, convolutional filters of sizes 1 × 1, 3 × 3, and 5 × 5, together with a 3 × 3 max-pooling operation, are applied in parallel, and the resulting feature maps are concatenated and forwarded to the subsequent layer. A key component for reducing the computational cost of the architecture is the dimensionality-reduction mechanism. As shown in Figure 7, 1 × 1 convolutional filters placed before the 3 × 3 and 5 × 5 convolutional layers reduce the number of channels in the feature maps, thereby alleviating computational bottlenecks. To facilitate gradient propagation during the training of the deep network, auxiliary classifiers are integrated into intermediate layers of the GoogLeNet architecture. These auxiliary branches are positioned after the Inception 4a and 4d modules and contribute additional supervision during training.

### 5.3. Fusion Method

Human emotions are expressed through a wide range of behavioral and physiological cues, including facial expressions, tone of voice, speech patterns, physiological responses, and body movements. Consequently, relying on a single modality such as visual or auditory input may be insufficient for robust emotion recognition. Vision-based systems, for example, can be adversely affected by variations in illumination, changes in head pose, occlusions, and camera placement. Similarly, speech-based systems are sensitive to background noise, microphone quality, and speaker-dependent characteristics. Physiological signal-based systems, including those utilizing EEG, face additional technical challenges related to motion artifacts, low signal-to-noise ratio, and electrode placement consistency.

These modality-specific limitations motivate the integration of information from multiple sources. Multimodal fusion aims to exploit complementary information across modalities to enhance recognition accuracy, improve system reliability, and increase robustness across varying conditions and individuals. In MER systems, the fused information may include facial expressions, speech signals, physiological measurements, and, in some cases, textual or semantic content. Since emotional information can be conveyed through both verbal and non-verbal channels, multimodal integration provides a natural framework for emotion recognition.

#### 5.3.1. Decision-Level Fusion (DLF)

DLF represents a late integration strategy in MER systems, in which each modality undergoes independent feature extraction, model training, and classification before their outputs are combined at the decision stage. In contrast to feature-level fusion (FLF), which integrates modalities during feature representation, DLF operates on predicted outputs such as class probabilities, confidence scores, or ranked class lists produced by separate unimodal classifiers. The primary objective of this approach is to enhance decision reliability and robustness by leveraging complementary information across modalities. DLF is particularly advantageous when modalities differ in sampling rates, reliability, or data quality, as the outputs of independently trained unimodal classifiers can be combined without requiring the direct integration of their underlying feature representations. This characteristic is especially relevant in the present study, since EEG and speech signals differ substantially in signal structure, dimensionality, and noise characteristics.

**Figure 8:**
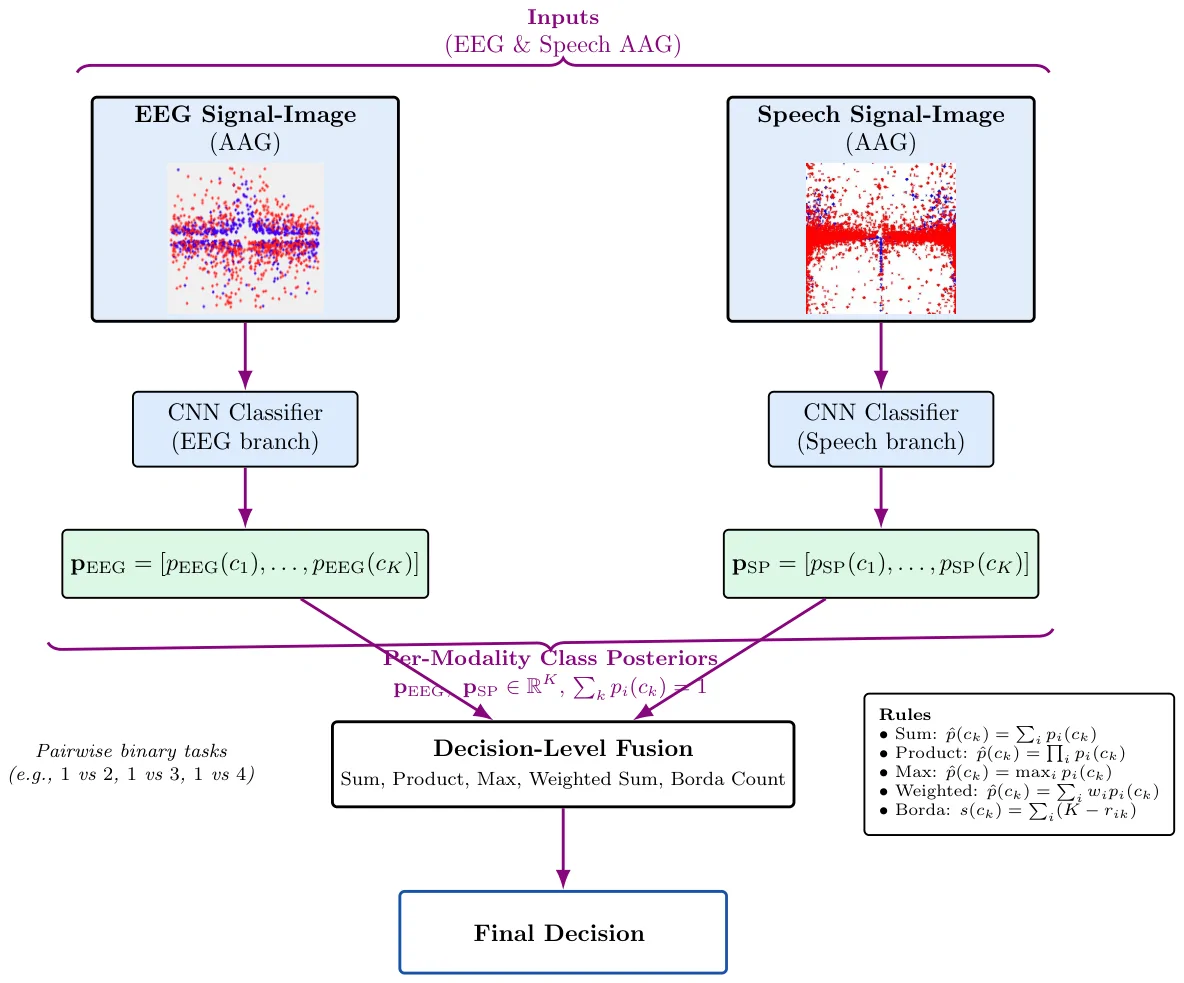
DLF pipeline integrating EEG and speech AAG inputs. Class probabilities from unimodal CNNs are combined using Sum, Product, Max, Weighted Sum, and Borda Count rules to obtain the final emotion decision.

In this study, several conventional DLF formulations, as shown in Figure 8, were implemented to combine the posterior outputs of the EEG and speech classifiers. These formulations represent different combination principles within the general classifier-fusion framework. However, under the present two-class, two-modality setting with normalized posterior probabilities, the Sum Rule (SR), Product Rule (PR), Max Rule (MR), and equal-weight Weighted Sum [WS(0.5)] yield the same hard-decision boundary. Therefore, these four formulations are interpreted in the present binary experiments as a decision-equivalent equal-weight fusion group rather than as four independent hard-decision strategies. The asymmetric WS configurations and Borda Count are discussed separately below.

##### (1) Sum Rule

The sum rule aggregates class probabilities across modalities by summing them. The class with the highest total probability is selected as the final decision:

(4) $$d\;=\;\arg\max_{k}\sum_{i=1}^{N}p_{i}\left(c_{k}\right)$$

where $p_{i}\left(c_{k}\right)$ denotes the probability assigned to class $c_{k}$ by the $i^{\mathrm{th}}$ modality, and *N* is the number of modalities. The sum rule is simple yet robust, as it balances the contribution of each modality.

##### (2) Product Rule

The product rule multiplies the probabilities predicted by each modality for a given class and selects the class with the highest product value:

(5) $$d\;=\;\arg\max_{k}\prod_{i=1}^{N}p_{i}\left(c_{k}\right)$$

In this formulation, $p_{i}\left(c_{k}\right)$ represents the class probability provided by modality *i*, while *N* denotes the total number of modalities. This rule assumes conditional independence among modalities and emphasizes consensus; if any modality assigns a very low probability to a class, the combined score decreases sharply.

In the present overt-speech recording setting, strict conditional independence between EEG and speech cannot be guaranteed, since residual speech-related muscular activity may contribute to the EEG signal. Therefore, the PR is evaluated here as an empirical fusion strategy, and its performance should not be interpreted as evidence that the conditional-independence assumption is strictly satisfied.

##### (3) Max Rule

The max rule considers the most confident prediction among all modalities for each class. The final decision corresponds to the class with the highest maximum probability:

(6) $$d\;=\;\arg\max_{k}\max_{i}\;p_{i}\left(c_{k}\right)$$

Here, $\max_{i}p_{i}\left(c_{k}\right)$ represents the maximum confidence assigned to class $c_{k}$ by any modality, and *d* denotes the selected class. This approach is particularly useful when one modality is significantly more reliable than the others, as it prioritizes the strongest signal for each class.

##### (4) Weighted Sum Rule

The WS rule allows the relative contributions of the EEG and speech modalities to be explicitly controlled during DLF. In the present implementation, α denotes the weight assigned to the EEG posterior probability, whereas (1−α) denotes the weight assigned to the speech posterior probability. The final decision is therefore defined as

(7) $$d\;=\;\arg\max_{k}\left[\alpha \;p_{\mathrm{EEG}}\left(c_{k}\right)+\left(1-\alpha \right)\;p_{\mathrm{Speech}}\left(c_{k}\right)\right],\;\alpha \in \left\{0.3,0.5,0.7\right\}.$$

Here, $p_{\mathrm{EEG}}\left(c_{k}\right)$ and $p_{\mathrm{Speech}}\left(c_{k}\right)$ represent the posterior probabilities assigned to class $c_{k}$ by the EEG and speech classifiers, respectively. Accordingly, α=0.3 corresponds to EEG/speech weights of 0.3/0.7, α=0.5 corresponds to equal weights of 0.5/0.5, and α=0.7 corresponds to EEG/speech weights of 0.7/0.3. Thus, WS(0.3) gives greater weight to the speech modality, whereas WS(0.7) gives greater weight to the EEG modality. Under the present binary two-modality setting with normalized posterior probabilities, WS(0.5) is decision-equivalent to the SR, PR, and MR at the hard-decision level.

##### (5) Borda Count Rule

The Borda Count (BC) method is a rank-based fusion strategy that combines decisions from multiple modalities by considering the ranking of classes rather than their posterior probability magnitudes. Each classifier assigns a rank $r_{ik}$ to each class $c_{k}$, and the corresponding Borda scores are aggregated as

(8) $$s\left(c_{k}\right)\;=\;\sum_{i=1}^{N}\left(K-r_{ik}\right),\;d\;=\;\arg\max_{k}s\left(c_{k}\right),$$

where *K* denotes the total number of classes, $r_{ik}$ represents the rank assigned to class $c_{k}$ by modality *i*, *N* is the number of modalities, and $s(c_{k})$ is the cumulative Borda score. The final decision *d* corresponds to the class with the highest aggregated score. Unlike probability-based fusion rules, BC operates on rank information and therefore does not directly depend on the magnitude of the posterior probabilities.

In the present binary two-modality setting, however, BC has a specific limitation. When the EEG and speech classifiers assign opposite rankings to the two classes, the aggregated Borda scores of the two classes can become equal. In the implemented decision rule, such ties are deterministically assigned to the second class of the corresponding binary pair because the final class decision uses a greater-than-or-equal comparison. Therefore, BC is retained in this study as a conventional rank-based reference formulation, but its results are interpreted cautiously and are not considered evidence of an independently superior fusion mechanism under the present binary setting.

## 6. Results and Discussion

The classification performances obtained from the unimodal and multimodal experiments are presented in this section. The proposed framework was evaluated on the newly developed KTU-MEDAFE dataset, which comprises synchronized speech, EEG, and facial recordings. Within the scope of the present experiments, EEG and speech signals were converted into image-based representations using the AAG transformation method and subsequently processed using deep CNN architectures. This unified representation enabled both modalities to be evaluated within the same image-based classification framework.

To establish baseline performance, unimodal classification experiments were first conducted separately for EEG- and speech-derived image representations using ResNet-50 and GoogLeNet. These architectures offer distinct architectural characteristics: ResNet-50 employs residual connections that facilitate the training of deeper networks and support robust feature learning [35], whereas GoogLeNet uses Inception modules to capture multi-scale visual patterns with relatively efficient computational complexity [36]. The use of two different architectures also enabled the consistency of the observed classification patterns to be examined across different CNN backbones. Subsequently, to examine the contribution of multimodal integration, DLF strategies were applied by combining the outputs of the unimodal classifiers. Conventional DLF methods were employed to evaluate the contribution of combining physiological and acoustic information under the subject-dependent experimental setting.

The classification performance of the proposed unimodal and multimodal frameworks was quantitatively evaluated using accuracy (Acc), as defined in Equation (9). The metric was computed based on the confusion matrix components, namely true positive (TP), true negative (TN), false positive (FP), and false negative (FN). Accuracy represents the overall proportion of correctly classified samples and is defined as

(9) $$\mathrm{Accuracy}\;=\;\frac{TP+TN}{TP+TN+FP+FN}$$

### 6.1. ResNet-50 Performance Analysis

The average classification accuracies obtained using the ResNet-50-based framework across 20 subjects are presented in Table 6. The table summarizes the mean performances of unimodal EEG, unimodal speech, and multimodal DLF models for all binary emotion pairs, whereas the detailed subject-wise best fusion performances together with the corresponding channel and fusion strategy selections are presented in Table 7. This two-level presentation enables both average model behavior and subject-specific modality interactions to be examined simultaneously.

The results indicate that the proposed multimodal fusion approach consistently outperforms both unimodal EEG and unimodal speech models across all pairwise comparisons. The average fusion accuracy ranges from 64.51% to 70.56%, whereas unimodal EEG and speech accuracies remain mostly between 54% and 62%. Among all binary tasks, the highest multimodal performance is achieved for the 2 vs. 4 pair with an average accuracy of 70.56%. Similarly, the 1 vs. 2 and 1 vs. 3 pairs also yield relatively strong fusion performances with accuracies of 68.84% and 67.88%, respectively, whereas relatively lower but still improved fusion accuracies are observed for the 1 vs. 4 and 3 vs. 4 pairs. This pattern suggests that the contribution of multimodal fusion is consistent across emotion pairs, although the absolute classification difficulty differs depending on the pair being evaluated.

These average tendencies are also reflected in the detailed subject-level findings reported in Table 7, where several subjects reach substantially higher pair-specific fusion accuracies. In unimodal evaluations, EEG and speech exhibit comparable performance levels, although their relative contribution varies depending on the emotion pair. For instance, EEG slightly outperforms speech in the 1 vs. 4 comparison, whereas speech achieves higher accuracies in 1 vs. 3, 2 vs. 3, and 2 vs. 4. This pair-dependent variation indicates that neither EEG nor speech acts as a uniformly dominant modality across all emotional comparisons. Instead, the discriminative contribution of each modality changes according to the emotional pair under consideration. Despite these pair-dependent differences, neither unimodal modality reaches the performance level obtained after fusion. This observation indicates that EEG and speech provide complementary emotional information, and their integration generally provides higher average classification performance. Compared with unimodal baselines, the multimodal fusion model provides an average improvement of approximately 6.4–10.2 percentage points across all emotion pairs. In addition to achieving higher accuracy, the fusion results also exhibit more consistent performance across different class pairs, suggesting improved robustness within the subject-dependent evaluation setting. Therefore, the ResNet-50 results support the main hypothesis of this study: decision-level integration of EEG and speech can compensate for modality-specific limitations and provide a more reliable classification outcome than either modality alone.

In addition to average performance analysis, subject-based classification results were examined to evaluate the effectiveness of decision-level fusion strategies under individual conditions. Among the first 25 recorded subjects, 20 subjects with usable EEG and speech recordings were included in the final analysis, whereas subjects with signal-related recording problems were excluded. The fusion accuracies obtained using Sum Rule (SR), Product Rule (PR), Max Rule (MR), Weighted Sum (WS), and Borda Count (BC) methods were comparatively evaluated to determine under which subject-specific conditions multimodal fusion provides the highest performance gain. As shown in Table 7, the optimal fusion method varies across subjects and pairwise emotion combinations, indicating that modality interaction remains highly subject-dependent. This variability also suggests that a single fixed fusion strategy may not be universally optimal for all subjects or emotion pairs, which is an important consideration for future subject-adaptive MER systems.

The fusion-strategy results should be interpreted in light of the analytical properties of the present binary two-modality setting. SR, PR, MR, and WS(0.5) share the same hard-decision boundary and therefore frequently appear jointly among the best-performing configurations in Table 7. Their joint occurrence should not be interpreted as evidence of four independently superior fusion mechanisms. In contrast, WS(0.3) and WS(0.7) represent asymmetric modality-weighting configurations, emphasizing speech and EEG, respectively. Their frequent selection in several subject-pair combinations indicates that unequal modality contributions can be beneficial under subject-dependent conditions. BC was retained as a rank-based reference formulation; however, because disagreement between the two modalities may produce tied Borda scores in the binary setting, its results are interpreted cautiously. Channel-based observations further indicate that no single EEG channel dominates all pair combinations. Nevertheless, Ch1, Ch2, and Ch4 appear more frequently in best-performing combinations, suggesting that these channels provide relatively stronger discriminative information under the ResNet-50 architecture. This finding supports the interpretation that both the selected EEG channel and the fusion strategy influence the effectiveness of multimodal decision integration. The frequent selection of weighted fusion rules further implies that EEG and speech contributions are not always equally informative. Instead, performance improves when the fusion mechanism allows one modality to contribute more strongly depending on the subject and class pair.

**Figure 9:**
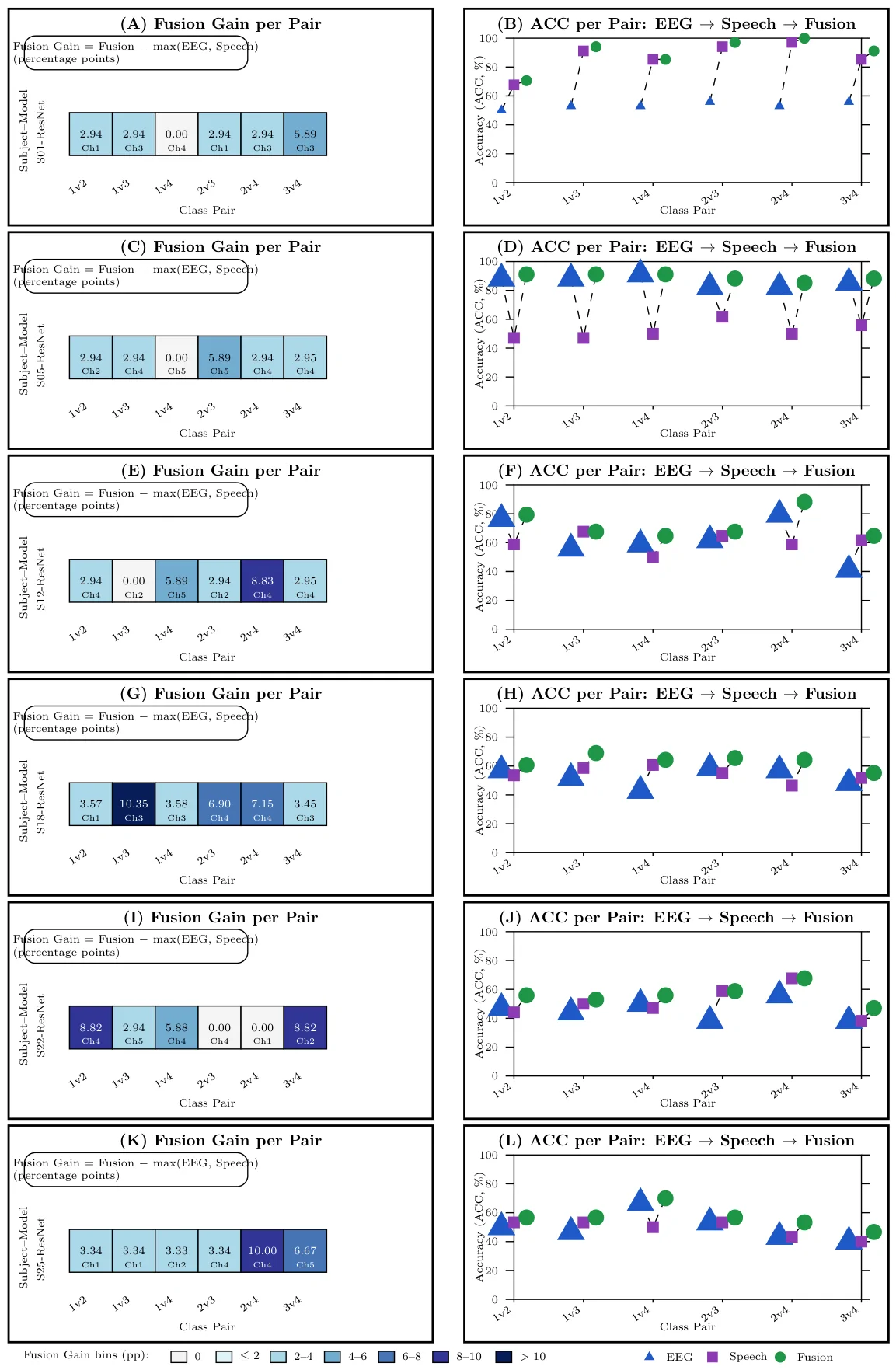
Subject-level multimodal fusion summary for ResNet-50. **Left** panels show fusion gain per class pair (Fusion − max(EEG, Speech), percentage points) together with the selected EEG channel (Ch1–Ch5). **Right** panels present EEG, Speech, and Fusion accuracy values.

The subject-level results reveal considerable inter-subject variability. In the ResNet-50-based experiments, the strongest performances are observed for subjects S01, S05, and S12 as seen in Figure 9. Subject S01 represents the most prominent example of multimodal complementarity. In particular, a fusion accuracy of 100% is achieved in the 2 vs. 4 pair, while several other comparisons such as 1 vs. 3, 2 vs. 3, and 3 vs. 4 also exceed 90%. However, this peak result should be interpreted as a subject- and pair-specific outcome rather than a general performance level across the dataset. A similar pattern is observed for subject S05, where EEG-based classification remains consistently strong across nearly all class pairs. Fusion results exceed 90% in most comparisons, especially under weighted sum strategies using higher EEG contribution coefficients. Subject S12 also demonstrates strong multimodal behaviour, particularly in the 2 vs. 4 comparison where fusion reaches 88.24%. These high-performing cases indicate that when at least one modality contains strong discriminative information and the second modality provides complementary support, decision-level fusion can substantially enhance classification performance.

In contrast, subjects S18, S22, and S25 exhibit comparatively lower fusion accuracies. For these subjects, unimodal EEG and speech performances remain relatively balanced at moderate levels, limiting the degree of complementary gain obtained after fusion. This suggests that fusion is most beneficial when the two modalities provide complementary rather than uniformly weak or redundant information. Overall, subject-level findings confirm that while ResNet-50-based multimodal fusion generally improves emotion recognition performance, the magnitude of improvement strongly depends on subject-specific signal characteristics, modality complementarity, selected channels, and the applied fusion strategy.

### 6.2. GoogLeNet Performance Analysis

The GoogLeNet-based average classification accuracies obtained from 20 subjects are presented in Table 8. The table summarizes the mean performance of unimodal EEG, unimodal speech, and multimodal fusion models across all pairwise emotion combinations, whereas detailed subject-wise best fusion performances are reported in Table 9. Similar to the ResNet-50 analysis, this two-level evaluation enables both overall classification tendencies and subject-specific fusion behavior to be examined under the GoogLeNet architecture.

An examination of the results reveals that the proposed multimodal fusion approach consistently provides higher classification accuracy than both unimodal EEG and unimodal speech models across all pairs. The average fusion accuracy ranges from 60.44% to 67.63%. The highest average fusion accuracy is achieved for the 2 vs. 4 pair (67.63%), followed closely by the 2 vs. 3 pair (67.41%). Comparable performances are also observed for the 1 vs. 2 and 1 vs. 3 pairs, whereas relatively lower fusion accuracies are obtained for the 1 vs. 4 and 3 vs. 4 pairs. This pattern indicates that multimodal fusion remains beneficial under GoogLeNet, although the absolute accuracy values are slightly lower than those obtained with the ResNet-50-based framework. The relatively stronger results for 1 vs. 2, 2 vs. 3, and 2 vs. 4 suggest that these emotion pairs contain more separable signal-image patterns, while the lower accuracies for 1 vs. 4 and 3 vs. 4 indicate more challenging class boundaries under the GoogLeNet feature representation.

In unimodal evaluations, EEG-based accuracies generally remain within the range of approximately 53–58%, while speech-based accuracies vary between approximately 54–62%. Although speech-based classification yields slightly higher results than EEG in several pairs, the performance of both unimodal modalities remains below the multimodal fusion results. This finding shows that, as in the ResNet-50 experiments, neither EEG nor speech alone provides a consistently dominant representation across all emotion pairs. Instead, the relative contribution of each modality changes depending on the pairwise emotional comparison. The consistent improvement obtained after fusion supports the interpretation that EEG and speech provide complementary decision evidence even when their individual classification performances remain moderate.

In addition to average accuracies, the detailed subject-wise best fusion performances presented in Table 9 further confirm that multimodal gains vary according to subject-specific modality characteristics, selected channels, and fusion strategies. Consistent with the analytical properties described in Section 5.3, SR, PR, MR, and WS(0.5) are interpreted as a decision-equivalent equal-weight group in the present binary two-modality experiments. The more informative differences in the current setting arise from the asymmetric WS configurations, particularly a=0.3 and a=0.7, which emphasize speech and EEG, respectively. Their repeated selection across subjects suggests that the relative contribution of the two modalities may vary according to subject and emotion pair. BC is retained as a rank-based reference formulation but is interpreted cautiously because of the tie behavior that can arise in the present two-class, two-modality setting. Channel-based analyses further reveal that channels Ch2, Ch3, and Ch4 provide relatively higher accuracy in both unimodal and multimodal settings. In multimodal fusion results, Ch2 and Ch4 appear as the most informative channels, suggesting that these channels contain more discriminative and complementary information for emotion recognition. This observation indicates that the effectiveness of multimodal fusion is influenced not only by the selected fusion rule but also by the EEG channel from which the physiological representation is derived. Therefore, the GoogLeNet results further support the importance of channel-dependent analysis in EEG-supported MER.

**Figure 10:**
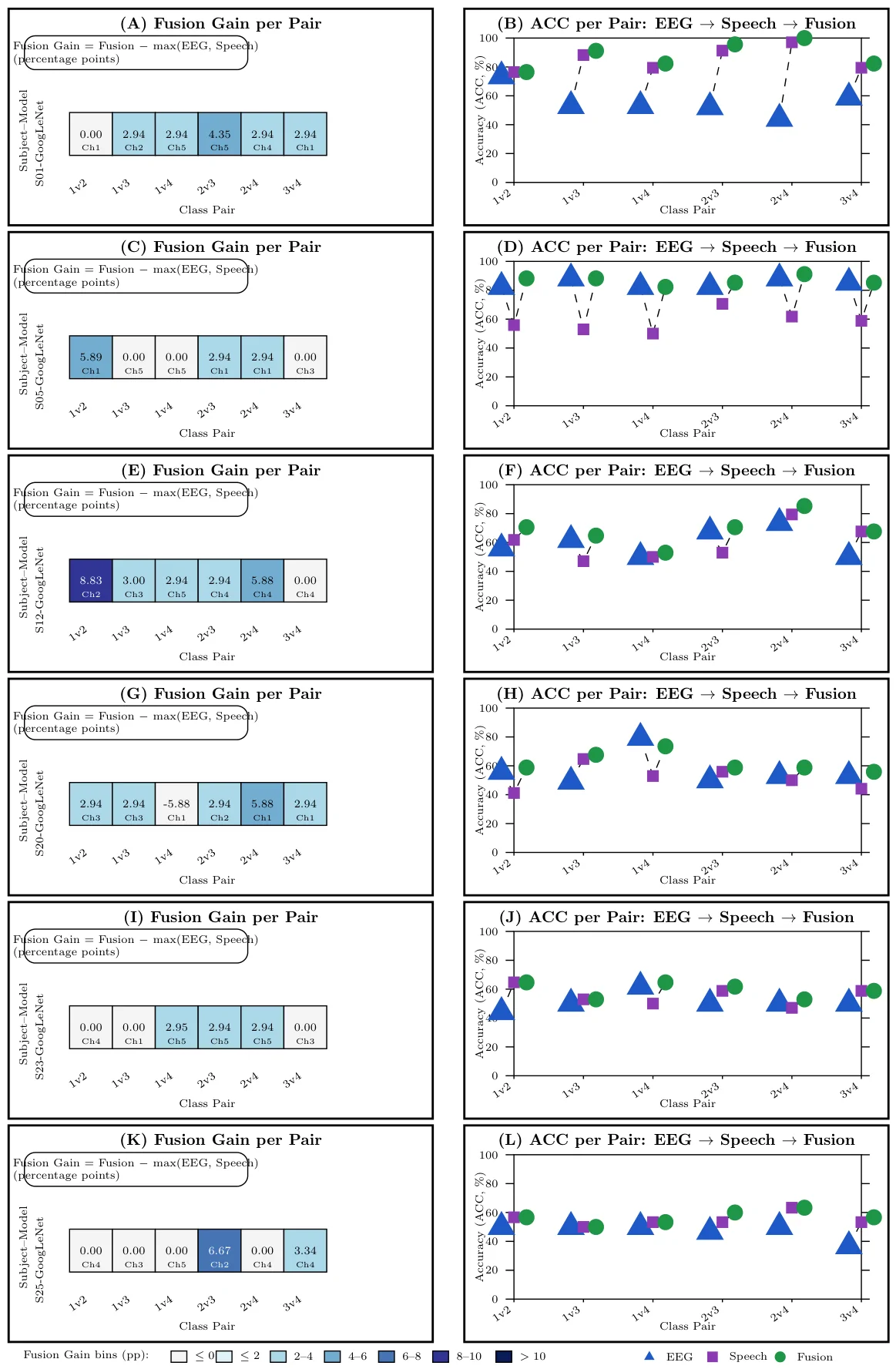
Subject-level multimodal fusion summary for GoogLeNet. **Left** panels show fusion gain per class pair (Fusion − max(EEG, Speech), percentage points) along with the selected channel (Ch1–Ch5). **Right** panels show EEG (triangle), Speech (square), and Fusion (circle) accuracy values. Top three subjects: S01, S05, S12. Bottom three subjects: S20, S23, S25.

The best classification performances were again achieved by subjects S01, S05, and S12 as seen in Figure 10. Subject S01 again emerges as one of the most successful cases under the GoogLeNet architecture. In particular, S01 achieves very high fusion performance in several emotion pairs, including the 2 vs. 4 comparison, where the fusion accuracy reaches 100%. As in the ResNet-50 analysis, this value should be interpreted as a subject- and pair-specific peak performance rather than a generalizable dataset-wide accuracy level. Similarly, subject S05 exhibits consistently high fusion accuracies. Subject S12 also maintains strong performance under GoogLeNet. The repeated appearance of S01, S05, and S12 among the best-performing subjects across both CNN architectures suggests that these subjects may contain clearer or more stable emotional patterns in at least one modality, allowing fusion to exploit modality complementarity more effectively. In contrast, subjects S20, S23, and S25 exhibit comparatively lower fusion accuracies. For these subjects, the limited improvement after fusion suggests that multimodal integration cannot fully compensate when both unimodal predictions are weak, unstable, or insufficiently complementary. Overall, the GoogLeNet-based multimodal framework provides stable improvements over unimodal models and confirms that subject-dependent variability remains one of the key factors affecting MER performance.

### 6.3. Comparison with Previous Multimodal Emotion Recognition Studies

Overall, the results indicate that multimodal fusion provides a meaningful contribution to emotion recognition performance compared with unimodal approaches. Across both ResNet-50 and GoogLeNet architectures, combining EEG and speech modalities generally improves classification accuracy, confirming the effectiveness of DLF. However, the obtained performance remains subject-dependent, and for certain subject-pair combinations where classification is particularly challenging, the achieved accuracies remain relatively lower despite multimodal integration. The use of two different deep learning models together with multiple fusion strategies demonstrates that multimodal approaches offer a consistent performance advantage, although the magnitude of improvement varies depending on modality complementarity, channel characteristics, and subject-specific signal properties. Therefore, the main contribution of the present results should not be interpreted solely in terms of absolute accuracy values, but also in terms of the consistent performance gain obtained through EEG–speech integration across different architectures, fusion rules, and emotion pairs.

In addition, the detailed comparative overview presented in Table 10 clearly indicates that performance levels reported in MER studies vary considerably depending on dataset characteristics, modality combinations, and adopted fusion strategies. Even within the same research domain, substantial differences in reported performance can be observed across datasets and experimental settings. For example, on the RECOLA dataset, Ortega et al. [9] reported a concordance correlation coefficient of approximately 0.749, whereas Ringeval et al. [10] achieved a higher value of 0.804 under a similar continuous emotion prediction framework. Likewise, on the IEMOCAP dataset, Yoon et al. [8] obtained a weighted accuracy of 71.8%, while Braunschweiler et al. [7] reached 85.50% by employing more advanced deep learning architectures integrating convolutional, recurrent, and transformer-based representations. In contrast, Avots et al. [4] reported an accuracy of 22.40% across heterogeneous datasets such as SAVEE, eNTERFACE’05, RML, and AFEW, thereby highlighting the substantial impact of dataset variability and cross-corpus complexity on achievable performance.

In light of these findings, direct numerical comparisons across MER studies should be interpreted with caution, since dataset composition, recording conditions, emotional annotation schemes, modality configurations, and evaluation protocols all play a decisive role in determining classification outcomes. This issue is particularly important for the present study, because KTU-MEDAFE is not a standard benchmark dataset with previously optimized protocols; rather, it is a newly constructed multimodal dataset designed to evaluate Turkish emotional speech together with EEG and facial recordings under controlled elicitation conditions. In this respect, the present study differs from many existing works by employing a newly constructed multimodal framework that integrates EEG and speech signals and transforms both modalities into image-based representations prior to classification. Unlike many audiovisual studies that rely mainly on facial and speech cues, the proposed framework incorporates a physiological modality, which introduces additional variability but also provides access to internal affective responses that may not be fully observable from external behavior alone. Consequently, the proposed framework operates under a more challenging and distinct evaluation setting than many audiovisual-based studies conducted on acted datasets.

Another important aspect of the present comparison is that high performance values reported in previous studies are often obtained under different assumptions, including different numbers of subjects, different emotion taxonomies, different modality combinations, and different train–test protocols. Therefore, the accuracy values obtained on KTU-MEDAFE should be interpreted within the context of a newly collected, language-specific, and subject-dependent multimodal dataset rather than as a direct one-to-one ranking against established benchmark results. Considering these factors, the obtained ResNet-50 and GoogLeNet results indicate that the proposed framework achieves competitive performance while addressing a more difficult and less explored EEG–speech fusion scenario.

Considering these findings together with the existing literature, the performance levels obtained in this study fall within a competitive and acceptable range when evaluated alongside the broad spectrum of results reported in MER studies. More importantly, the consistent improvements achieved through multimodal fusion across both ResNet-50 and GoogLeNet architectures confirm the complementary nature of EEG and speech modalities. Furthermore, the systematic evaluation of two distinct deep learning backbones together with multiple DLF strategies provides a comprehensive assessment of robustness, subject variability, and methodological generalizability. These results suggest that KTU-MEDAFE can serve as a useful resource for future studies investigating language-specific MER, particularly those focusing on EEG–speech complementarity, subject-dependent variability, and DLF strategies.

A further limitation concerns the acquisition of EEG during overt speech. Although the same preprocessing protocol was consistently applied across subjects and emotion categories, residual speech-related muscular and ocular activity cannot be completely excluded from the EEG recordings. Consequently, part of the discriminative EEG information may reflect activity associated with articulation in addition to neural responses related to emotional processing. The EEG and EEG–speech fusion results should therefore be interpreted with this potential confounding factor in mind.

## 7. Conclusions and Future Work

MER has attracted increasing attention in recent years; however, achieving robust and reliable performance across different subjects and emotional states remains a significant challenge. In this study, we introduced KTU-MEDAFE, a newly developed Turkish multimodal emotion dataset containing synchronized EEG, speech, and facial recordings, and evaluated an EEG–speech DLF framework using this dataset. EEG and speech signals were transformed into image-based representations and classified using two deep learning architectures, ResNet-50 and GoogLeNet. In addition to unimodal evaluations, several conventional DLF formulations, including equal-weight score-based fusion, asymmetric weighted fusion, and rank-based fusion, were examined. Under the present binary two-modality setting, SR, PR, MR, and WS(0.5) were analytically equivalent at the hard-decision level and were therefore interpreted as a decision-equivalent equal-weight group rather than as independent fusion mechanisms.

The experimental results indicate that the proposed multimodal framework generally provides higher average classification performance than the unimodal EEG- and speech-based models across the evaluated binary emotion pairs. Across both architectures, multimodal fusion provides consistent improvements in classification accuracy, although the magnitude of these improvements varies depending on the subject, emotion pair, EEG channel, model architecture, and fusion strategy. These findings indicate that EEG and speech provide complementary information for emotion recognition while also confirming that subject-dependent variability remains a major challenge for MER.

Overall, this study demonstrates the potential of KTU-MEDAFE as a language-specific multimodal resource and establishes a solid baseline for future research on EEG–speech fusion, subject-adaptive modeling, and robust MER systems. Although this study establishes initial baseline results on the KTU-MEDAFE dataset using AAG-based signal-to-image representations and decision-level EEG–speech fusion, it is not intended to provide an exhaustive comparison of all EEG, speech, and multimodal fusion methods. A further limitation concerns the lower elicitation consistency observed for the funny category. Future studies using KTU-MEDAFE may therefore consider filtering trials according to self-assessment scores or excluding this category from specific analyses when stricter stimulus response agreement is required. Future work will extend the current framework by incorporating conventional EEG features, time frequency representations, MFCCs, log-Mel spectrograms, modern speech embeddings, and more advanced multimodal fusion architectures, including transformer-based models. The evaluation protocol will also be repeated using multiple random seeds to examine the stability of classification performance across different data partitions and model initializations, while alternative EEG preprocessing and source-separation strategies, together with high-frequency power analyses and temporally aligned speech-activity analyses, will be investigated to more systematically assess possible speech-related myogenic contributions to the EEG recordings. In addition, future studies will evaluate cross-subject generalization and integrate the facial video modality with EEG and speech to develop a complete trimodal emotion recognition framework, and will further improve the accessibility and usability of the KTU-MEDAFE dataset.

## Figures and Tables

**Table 1:** Summary Statistics of the Proposed KTU-MEDAFE Dataset.

Attribute	Description
No. of subjects	40 participants (26 male, 14 female)
Sessions	39 subjects completed 2 sessions; 1 subject completed only 1 session
Recordings per subject	172 trials per session; 344 trials per subject for each modality
EEG signals	5 channels (AF3, AF4, T7, T8, Pz); sampling rate: 128 Hz
Face videos	Recorded for all participants; one subject excluded from public sharing consent
Age range	19–38 years (mean ≈ 28.5)
Visual modality	RGB videos, 1920 × 1080 resolution at 30 fps

**Table 2:** Composition of Text-Based Emotional Stimuli in the KTU-MEDAFE Dataset.

Category	Details
No. of texts	172 per session (43 per category), 344 in total
Language	Turkish
Stimulus categories	Funny, Surprising, Sad, Neutral
Funny class	Turkish jokes and humorous anecdotal texts
Surprising class	Unusual factual events and Guinness World Records examples
Sad class	Emotionally negative texts involving loss or difficult social events
Neutral class	Emotion-neutral factual sentences prepared with AI-assisted support
Rating value	1 to 9 (participant self-assessment after each trial)

**Table 3:** Self-assessment results for emotional label validation in the first paradigm after excluding S06, S11, S15, S19 and S24.

Emotion Label	Code	Trials	Participants	Ratings	Mean ± SD	Ratings ≥ 6 (%)	Ratings ≤ 3 (%)
Neutral	1	43	20	860	7.64 ± 1.73	88.15	4.10
Funny	2	43	20	860	5.29 ± 2.42	52.82	28.46
Surprising	3	43	20	860	6.65 ± 1.96	76.41	9.19
Sad	4	43	20	860	6.71 ± 1.99	77.85	11.30
Overall	–	172	20	3440	6.57 ± 2.20	73.81	13.26

**Table 4:** Self-assessment results for emotional label validation in the second paradigm after excluding S06, S11, S15, S19 and S24.

Emotion Label	Code	Trials	Participants	Ratings	Mean ± SD	Ratings ≥ 6 (%)	Ratings ≤ 3 (%)
Neutral	1	43	20	860	7.80 ± 1.53	90.59	2.21
Funny	2	43	20	860	4.96 ± 2.29	46.07	30.45
Surprising	3	43	20	860	6.65 ± 1.85	78.18	8.42
Sad	4	43	20	860	6.70 ± 1.96	78.18	10.52
Overall	–	172	20	3440	6.53 ± 2.18	73.26	12.90

**Table 5:** Descriptive statistics of utterance duration and local-maxima count by emotion category and paradigm.

Paradigm	Emotion	N Utterances	Duration (s), Mean ± SD	Local Maxima, Mean ± SD
First	Neutral	860	10.08 ± 1.51	62,203 ± 12,379
First	Funny	860	10.33 ± 1.80	66,710 ± 14,841
First	Surprising	860	10.49 ± 1.54	65,178 ± 13,785
First	Sad	860	10.81 ± 1.61	67,976 ± 14,321
Second	Neutral	860	10.30 ± 1.59	64,764 ± 12,925
Second	Funny	860	11.34 ± 1.90	75,324 ± 17,098
Second	Surprising	860	10.38 ± 1.54	65,848 ± 13,520
Second	Sad	860	10.48 ± 1.40	66,550 ± 12,589

**Table 6:** Average classification accuracy (%) using ResNet50-based unimodal and multimodal approaches.

Pair	Avg. EEG	Avg. Speech	Avg. Fusion
1 vs. 2	58.29	58.67	68.84
1 vs. 3	55.50	60.52	67.88
1 vs. 4	58.69	55.37	65.13
2 vs. 3	54.95	59.83	66.67
2 vs. 4	59.61	61.67	70.56
3 vs. 4	54.44	54.18	64.51

**Table 7:** Subject-wise best-performing fusion configurations under the predefined evaluation protocol using ResNet-50.

**Subject**	**1 vs. 2**				**1 vs. 3**				**1 vs. 4**			
	**EEG**	**Audio**	**Fusion**	**Method**	**EEG**	**Audio**	**Fusion**	**Method**	**EEG**	**Audio**	**Fusion**	**Method**
S01	50.00	67.65	70.59	SR/PR/MR/WS(0.5)-Ch1	52.94	91.18	94.12	SR/PR/MR/WS(0.5)-Ch3	52.94	85.29	85.29	WS(0.3)-Ch4
S02	61.76	70.59	79.41	SR/PR/MR/WS(0.5)-Ch2	64.71	58.82	67.65	BC-Ch2	64.71	61.76	70.59	SR/PR/MR/WS(0.5)-Ch1
S03	47.06	67.65	73.53	SR/PR/MR/WS(0.5)-Ch3	50.00	50.00	61.76	SR/PR/MR/WS(0.5)-Ch1	64.71	55.88	64.71	WS(0.3)-Ch4
S04	58.82	55.88	70.59	SR/PR/MR/WS(0.5)-Ch5	55.88	64.71	67.65	SR/PR/MR/WS(0.3/0.5)-Ch5	61.76	52.94	67.65	WS(0.7)-Ch4
S05	88.24	47.06	91.18	WS(0.7)-Ch2	88.24	47.06	91.18	WS(0.7)-Ch4	91.18	50.00	91.18	WS(0.7)-Ch5
S07	50.00	61.76	61.76	WS(0.3)-Ch4	55.88	55.88	61.76	WS(0.7)-Ch1	51.43	51.43	51.43	All Methods-Ch5
S08	55.88	58.82	61.76	All excluding WS(0.3)-Ch3	61.76	52.94	64.71	SR/PR/MR/WS(0.5)-Ch2	61.76	50.00	64.71	BC-Ch1
S09	61.76	50.00	73.53	SR/PR/MR/WS(0.5)-Ch2	58.82	50.00	61.76	WS(0.3)-Ch3	50.00	50.00	52.94	WS(0.7)-Ch4
S10	55.88	52.94	73.53	SR/PR/MR/WS(0.5)-Ch4	47.06	58.82	70.59	SR/PR/MR/WS(0.5)-Ch2	58.82	52.94	61.76	SR/PR/MR/WS(0.5/0.7)-Ch2
S12	76.47	58.82	79.41	WS(0.7)-Ch4	55.88	67.65	67.65	WS(0.3)/BC-Ch2	58.82	50.00	64.71	WS(0.7)-Ch5
S13	61.54	57.69	65.38	BC-Ch1	61.54	69.23	73.08	BC-Ch1	60.00	60.00	64.00	SR/PR/MR/WS(0.5)-Ch2
S14	73.53	64.71	82.35	SR/PR/MR/WS(0.5/0.7)-Ch2	50.00	64.71	70.59	WS(0.3)-Ch5	47.06	52.94	55.88	SR/PR/MR/WS(0.3/0.5)-Ch3
S16	50.00	67.65	67.65	WS(0.3)-Ch1	47.06	52.82	58.82	SR/PR/MR/WS(0.5)-Ch2	58.82	47.06	55.88	WS(0.7)/BC-Ch1
S17	52.94	61.76	64.71	SR/PR/MR/WS(0.5)-Ch2	52.94	70.59	61.76	WS(0.3)-Ch1	58.82	61.76	64.71	WS(0.3)-Ch3
S18	57.14	53.57	60.71	BC-Ch1	51.72	58.62	68.97	SR/PR/MR/WS(0.5)-Ch3	42.86	60.71	64.29	WS(0.3/0.7)-Ch3
S20	58.82	61.76	61.76	SR/PR/MR/WS(0.5)-Ch1	64.71	67.65	73.53	SR/PR/MR/WS(0.5)-Ch3	58.82	58.82	70.59	SR/PR/MR/WS(0.5)-Ch2
S21	50.00	50.00	55.88	SR/PR/MR/WS(0.5)-Ch4	50.00	64.71	64.71	All Methods-Ch5	61.76	52.94	61.76	BC-Ch5
S22	47.06	44.12	55.88	BC-Ch4	44.12	50.00	52.94	WS(0.3)-Ch5	50.00	47.06	55.88	WS(0.7)-Ch4
S23	58.82	67.65	70.59	WS(0.3)-Ch5	50.00	61.76	67.65	WS(0.3)-Ch4	52.94	55.88	64.71	SR/PR/MR/WS(0.5)-Ch1
S25	50.00	53.33	56.67	WS(0.3)-Ch1	46.67	53.33	56.67	WS(0.3)-Ch1	66.67	50.00	70.00	WS(0.7)-Ch2
**Subject**	**2 vs. 3**				**2 vs. 4**				**3 vs. 4**			
	**EEG**	**Audio**	**Fusion**	**Method**	**EEG**	**Audio**	**Fusion**	**Method**	**EEG**	**Audio**	**Fusion**	**Method**
S01	55.88	94.12	97.06	SR/PR/MR/WS(0.5)-Ch1	52.94	97.06	100.00	WS(0.3)-Ch3	55.88	85.29	91.18	SR/PR/MR/WS(0.3)-Ch3
S02	50.00	61.76	67.65	SR/PR/MR/WS(0.3)-Ch4	67.65	70.59	82.35	BC-Ch1	50.00	47.06	58.82	BC-Ch4
S03	55.88	44.12	61.76	WS(0.7)-Ch4	52.94	52.94	58.82	SR/PR/MR/WS(0.5)-Ch1	59.38	40.62	62.50	WS(0.7)-Ch4
S04	58.82	64.71	67.65	WS(0.3)-Ch2	71.76	76.47	79.41	WS(0.3)-Ch5	55.88	67.65	70.59	BC-Ch5
S05	82.35	61.76	88.24	MR/WS(0.7)-Ch5	82.35	50.00	85.29	SR/PR/MR/WS(0.5)-Ch4	85.29	55.88	88.24	WS(0.7)-Ch4
S07	61.76	58.82	61.76	WS(0.7)/BC-Ch1	61.11	61.11	66.67	BC-Ch5/Ch2	61.76	50.00	64.71	WS(0.7)-Ch1
S08	47.06	52.94	55.88	WS(0.3)-Ch4/Ch2	55.88	58.82	64.71	SR/PR/MR/WS(0.5)-Ch2	55.88	47.06	64.71	WS(0.7)-Ch3
S09	52.94	55.88	61.76	SR/PR/MR/WS(0.5)/BC-Ch3	70.59	67.65	73.53	WS(0.7)/BC-Ch1	55.88	58.82	61.76	WS(0.3)/BC-Ch1/Ch4
S10	58.82	64.71	67.65	SR/PR/MR/WS(0.5)-Ch2	55.88	58.82	67.65	WS(0.5)/BC-Ch5	61.76	50.00	73.53	SR/PR/MR/WS(0.5)-Ch1
S12	61.76	64.71	67.65	SR/PR/MR/WS(0.5)-Ch2	79.41	58.82	88.24	SR/PR/MR/WS(0.5)-Ch4	41.18	61.76	64.71	WS(0.3)-Ch4
S13	57.69	61.54	73.08	SR/PR/MR/WS(0.5)-Ch1	56.00	56.00	68.00	All Methods-Ch2	44.00	60.00	64.00	All Methods-Ch5
S14	52.94	76.47	79.41	SR/PR/MR/WS(0.5)-Ch3	52.94	76.47	76.47	SR/PR/MR/WS(0.3/0.5)-Ch3	58.82	55.88	61.76	BC-Ch5
S16	58.82	47.06	61.76	WS(0.7)-Ch2	55.88	47.06	55.88	WS(0.7)-Ch2	61.76	44.12	61.76	BC-Ch4
S17	47.06	50.00	58.82	SR/PR/MR/WS(0.5)-Ch1	58.82	58.82	61.76	BC-Ch2	58.82	58.82	64.71	BC-Ch3
S18	58.62	55.17	65.52	WS(0.7)-Ch4	57.14	46.43	64.29	WS(0.7)-Ch4	48.28	51.72	55.17	BC-Ch3
S20	55.88	52.94	58.82	BC-Ch5	52.94	61.76	67.65	SR/PR/MR/WS(0.5)-Ch3	64.71	47.06	64.71	WS(0.7)-Ch2
S21	50.00	58.82	61.76	SR/PR/MR/WS(0.5)-Ch1	47.06	61.76	64.71	WS(0.3)-Ch5	52.94	64.71	70.59	WS(0.3)-Ch3
S22	38.24	58.82	58.82	WS(0.3)-Ch4	55.88	67.65	67.65	SR/PR/MR/WS(0.5)-Ch1	38.24	38.24	47.06	BC-Ch2
S23	41.18	58.82	61.76	WS(0.3)-Ch2	61.76	61.76	64.71	All excluding WS(0.3)-Ch1	38.24	58.82	52.94	WS(0.3)-Ch5
S25	53.33	53.33	56.67	All excluding BC-Ch4	43.33	43.33	53.33	SR/PR/MR/WS(0.5)-Ch4	40.00	40.00	46.67	SR/PR/MR/WS(0.5)/BC-Ch5

*Note:* WS = Weighted Sum; SR = Sum Rule; PR = Product Rule; MR = Max Rule; BC = Borda Count. For WS, α denotes the EEG weight and 1−α denotes the speech weight. Under the present binary two-modality setting, SR, PR, MR, and WS(0.5) are equivalent at the hard-decision level.

**Table 8:** Average classification ACC (%) using GoogLeNet-based unimodal and multimodal approaches.

Pair	Avg. EEG	Avg. Speech	Avg. Fusion
1 vs. 2	57.68	57.98	67.26
1 vs. 3	55.94	58.92	63.85
1 vs. 4	52.55	53.50	61.24
2 vs. 3	56.63	61.49	67.41
2 vs. 4	55.42	61.91	67.63
3 vs. 4	54.12	56.43	60.44

**Table 9:** Subject-wise best-performing fusion configurations under the predefined evaluation protocol using GoogLeNet.

**Subject**	**1 vs. 2**				**1 vs. 3**				**1 vs. 4**			
	**EEG**	**Audio**	**Fusion**	**Method**	**EEG**	**Audio**	**Fusion**	**Method**	**EEG**	**Audio**	**Fusion**	**Method**
S01	73.53	76.47	76.47	SR/PR/MR/WS(0.3/0.5/0.7)-Ch1	52.94	88.24	91.18	SR/PR/MR/WS(0.5)-Ch2	52.94	79.41	82.35	BC-Ch5
S02	50.00	79.41	82.35	All excluding BC-Ch2	64.71	58.82	67.65	BC-Ch2	37.50	53.12	64.71	WS(0.3)-Ch3
S03	52.94	61.76	64.71	SR/PR/MR/WS(0.5)-Ch1	51.85	44.44	55.56	SR/PR/MR/WS(0.5)-Ch2	44.12	55.88	61.76	SR/PR/MR/WS(0.5)-Ch1
S04	64.71	47.06	61.76	SR/PR/MR/WS(0.5/0.7)-Ch2	50.00	76.47	61.76	BC-Ch2	44.12	47.06	52.94	WS(0.7)-Ch5
S05	82.35	55.88	88.24	WS(0.7)-Ch1	88.24	52.94	88.24	WS(0.7)-Ch5	82.35	50.00	82.35	All excluding BC-Ch5
S07	73.53	67.65	76.47	All excluding WS(0.3)-Ch4	58.33	55.56	63.89	BC-Ch4	60.00	51.43	60.00	WS(0.7)-Ch5
S08	50.00	50.00	73.53	SR/PR/MR/WS(0.5)-Ch1	64.71	50.00	52.94	All excluding WS(0.3)-Ch2	50.00	41.18	50.00	SR/PR/MR/WS(0.5)-Ch3
S09	55.88	61.76	67.65	BC-Ch5	47.06	64.71	67.65	WS(0.3)-Ch4	58.82	61.76	64.71	WS(0.3)-Ch5
S10	47.06	50.00	55.88	BC-Ch4	58.82	58.82	61.76	WS(0.3)-Ch1	41.18	47.06	52.94	SR/PR/MR/WS(0.5)-Ch3
S12	55.88	61.76	70.59	SR/PR/MR/WS(0.5)-Ch2	61.76	47.06	64.76	WS(0.7)-Ch3	50.00	50.00	52.94	WS(0.3)-Ch5
S13	65.38	50.00	65.38	All excluding BC-Ch1	53.85	61.54	57.69	All excluding BC-Ch2	36.00	48.00	52.00	SR/PR/MR/WS(0.5)-Ch5
S14	52.94	58.82	61.76	WS(0.7)/BC-Ch5	58.82	50.00	61.76	WS(0.3)-Ch3	50.00	58.82	61.76	WS(0.3)-Ch1
S16	47.06	58.82	61.76	SR/PR/MR/WS(0.5)-Ch5	50.00	50.00	50.00	All excluding WS(0.7)-Ch3	58.82	50.00	58.82	WS(0.7)/BC-Ch4
S17	70.59	64.71	73.53	BC-Ch5	58.82	55.88	61.76	SR/PR/MR/WS(0.5)-Ch2/Ch1	50.00	55.88	58.82	WS(0.7)-Ch1
S18	50.00	50.00	64.29	All Methods-Ch3	58.62	55.17	58.62	BC-Ch2	32.14	46.43	53.57	SR/PR/MR/WS(0.5)-Ch2
S20	55.88	41.18	58.82	WS(0.7)-Ch3	49.06	64.71	67.65	WS(0.3)-Ch3	79.41	52.94	73.53	WS(0.7)-Ch1
S21	61.76	47.06	64.71	WS(0.7)-Ch1	47.06	79.41	79.41	WS(0.3)/BC-Ch4	61.76	58.82	61.76	BC-Ch3
S22	50.00	55.88	55.88	All excluding BC-Ch3	44.12	61.76	61.76	WS(0.3)-Ch3	50.00	58.82	61.76	WS(0.3)-Ch2
S23	44.12	64.71	64.71	SR/PR/MR/WS(0.3/0.5)-Ch4	50.00	52.94	52.94	All Methods-Ch1	61.76	50.00	64.71	All excluding BC-Ch5
S25	50.00	56.67	56.67	All Methods-Ch4	50.00	50.00	50.00	WS(0.7)/BC-Ch3	50.00	53.33	53.33	All Methods-Ch5
**Subject**	**2 vs. 3**				**2 vs. 4**				**3 vs. 4**			
	**EEG**	**Audio**	**Fusion**	**Method**	**EEG**	**Audio**	**Fusion**	**Method**	**EEG**	**Audio**	**Fusion**	**Method**
S01	52.17	91.30	95.65	SR/PR/MR/WS(0.3/0.5)-Ch5	44.12	97.06	100.00	SR/PR/MR/WS(0.3)-Ch4	58.82	79.41	82.35	WS(0.3)/BC-Ch1
S02	56.67	80.00	83.33	SR/PR/MR/WS(0.5/0.7)-Ch2	50.00	76.47	76.47	All excluding BC-Ch2	50.00	58.82	58.82	WS(0.7)-Ch3
S03	51.85	44.44	55.56	SR/PR/MR/WS(0.5)-Ch2	61.54	65.38	65.38	All excluding WS(0.3)-Ch2	53.85	65.38	57.69	WS(0.3)-Ch5
S04	61.76	67.65	67.65	SR/PR/MR/WS(0.3/0.5)/BC-Ch3	65.38	55.88	73.08	WS(0.7)-Ch2	44.12	41.18	47.06	WS(0.3)-Ch4
S05	82.35	70.59	85.29	SR/PR/MR/WS(0.5/0.7)-Ch1	88.24	61.76	91.18	WS(0.7)-Ch1	85.29	58.82	85.29	WS(0.7)/BC-Ch3
S07	85.29	50.00	65.29	WS(0.7)-Ch1	55.88	41.18	58.82	WS(0.3/0.7)/BC-Ch1	61.76	38.24	41.76	BC-Ch2
S08	61.76	58.82	64.71	SR/PR/MR/WS(0.5)-Ch4	50.00	50.00	55.88	SR/PR/MR/WS(0.3/0.5)-Ch5	58.82	47.06	58.82	All excluding BC-Ch2
S09	50.00	73.53	73.52	All excluding BC-Ch5	52.94	61.76	64.71	SR/PR/MR/WS(0.3/0.5)-Ch4	50.00	61.76	61.76	All excluding BC-Ch1
S10	47.06	73.53	73.53	WS(0.3)-Ch3	61.76	55.88	67.65	SR/PR/MR/WS(0.5)-Ch5	50.00	55.88	55.88	BC-Ch2
S12	67.65	52.94	70.59	WS(0.7)-Ch4	73.53	79.41	85.29	WS(0.3)-Ch4	50.00	67.65	67.65	All Methods-Ch4
S13	50.00	61.54	61.54	All excluding BC-Ch4	52.00	60.00	60.00	All Methods-Ch4	60.00	64.00	64.00	All excluding BC-Ch4
S14	58.82	76.47	79.41	WS(0.3)-Ch3	47.06	79.41	79.41	WS(0.3)-Ch3	50.00	50.00	52.94	WS(0.5)-Ch3
S16	52.94	50.00	61.76	WS(0.7)-Ch2	50.00	61.76	61.76	All Methods-Ch2				
S17	55.88	47.06	55.88	SR/PR/MR/WS(0.5/0.7)-Ch2	52.94	50.00	55.88	SR/PR/MR/WS(0.5)-Ch2	50.00	64.71	64.71	All Methods-Ch2
S18	51.72	55.17	62.07	BC-Ch2	50.00	64.29	64.29	All Methods-Ch3	48.28	48.28	51.72	SR/PR/MR/WS(0.5)-Ch1
S20	50.00	55.88	58.82	SR/PR/MR/WS(0.3/0.5)-Ch2	52.94	50.00	58.82	WS(0.3)-Ch1	52.94	44.12	55.88	WS(0.7)-Ch1
S21	50.00	52.94	55.88	WS(0.7)-Ch1	50.00	52.94	52.94	All excluding WS(0.3)-Ch4	47.06	50.00	52.94	WS(0.3)/BC-Ch2
S22	50.00	55.88	55.88	All Methods-Ch1	50.00	64.71	64.71	WS(0.3/0.7)-Ch5	70.59	64.71	73.53	WS(0.7)-Ch2
S23	50.00	58.82	61.76	SR/PR/MR/WS(0.5/0.7)-Ch5	50.00	47.06	52.94	SR/PR/MR/WS(0.5)-Ch5	50.00	58.82	58.82	All excluding BC-Ch3
S25	46.67	53.33	60.00	BC-Ch2	50.00	63.33	63.33	All excluding BC-Ch4	36.67	53.33	56.67	SR/PR/MR/WS(0.5)-Ch4

*Note:* WS = Weighted Sum; SR = Sum Rule; PR = Product Rule; MR = Max Rule; BC = Borda Count. For WS, α denotes the EEG weight and 1−α denotes the speech weight. Under the present binary two-modality setting, SR, PR, MR, and WS(0.5) are equivalent at the hard-decision level.

**Table 10:** Summary of Multimodal Emotion Recognition Studies.

Study	Dataset	Fusion	Metric	Score
[1]	eNTERFACE	FLF	ACC	87.95%
[10]	RECOLA	FLF+DLF	CCC	A:0.804, V:0.528
[13]	AFEW	FLF + DLF	ACC	56.66%
[3]	eNTERFACE	DLF	ACC	89.39%
[8]	IEMOCAP	FLF	WAP	71.8%
[9]	RECOLA	FLF + DLF	CCC	A:0.749, V:0.565
[4]	SAVEE, eNTERFACE’05, RML, AFEW	DLF	ACC	22.40%
[16]	MED4	FLF + DLF	ACC	89.04%, 90.59%
[7]	IEMOCAP	FLF	UA	85.5%
[2]	eNTERFACE’05, RAVDESS	FLF	ACC	88.11%, 93.23%
[19]	CMU-MOSEI	DLF	ACC	85.85%
[20]	MELD, IEMOCAP	FLF	ACC	72.03%, 67.28%
[21]	CREMA-D, IEMOCAP	FLF	F1-micro, WA	0.817, 74.6%
This work	KTU-MEDAFE	DLF	ACC	70.56% (ResNet), 67.63% (GoogLeNet)

*Note:* FLF = Feature-Level Fusion; DLF = Decision-Level Fusion; ACC = Accuracy; CCC = Concordance Correlation Coefficient; WAP = Weighted Average Precision; UA = Unweighted Accuracy; WA = Weighted Accuracy; A = Arousal; V = Valence.

## Data Availability

The complete curated KTU-MEDAFE dataset will be made publicly available for research use upon the publication of this article, subject to the corresponding participant consent permissions. The data supporting the findings of this study are available at https://drive.google.com/drive/folders/10b-FabMlS4VL6Ttvah7iHmDGIRh16g73?usp=sharing (accessed on 20 August 2026).
